# Prohormone convertase 1/3 deficiency causes obesity due to impaired proinsulin processing

**DOI:** 10.1038/s41467-022-32509-4

**Published:** 2022-08-13

**Authors:** Daniel T. Meier, Leila Rachid, Sophia J. Wiedemann, Shuyang Traub, Kelly Trimigliozzi, Marc Stawiski, Loïc Sauteur, Denise V. Winter, Christelle Le Foll, Catherine Brégère, Raphael Guzman, Alex Odermatt, Marianne Böni-Schnetzler, Marc Y. Donath

**Affiliations:** 1 Clinic of Endocrinology, Diabetes and Metabolism, University Hospital Basel, Basel, Switzerland; 2 Department of Biomedicine, University of Basel, Basel, Switzerland; 3 Division of Molecular and Systems Toxicology, Department of Pharmaceutical Sciences, University of Basel, Basel, Switzerland; 4 Institute of Veterinary Physiology, University of Zurich, 8057 Zurich, Switzerland; 5 Department of Neurosurgery, University of Basel, Basel, Switzerland

**Keywords:** Obesity, Metabolic syndrome, Pre-diabetes, Obesity

## Abstract

Defective insulin processing is associated with obesity and diabetes. Prohormone convertase 1/3 (PC1/3) is an endopeptidase required for the processing of neurotransmitters and hormones. PC1/3 deficiency and genome-wide association studies relate PC1/3 with early onset obesity. Here, we find that deletion of PC1/3 in obesity-related neuronal cells expressing proopiomelanocortin mildly and transiently change body weight and fail to produce a phenotype when targeted to Agouti-related peptide- or nestin-expressing tissues. In contrast, pancreatic β cell-specific PC1/3 ablation induces hyperphagia with consecutive obesity despite uncontrolled diabetes with glucosuria. Obesity develops not due to impaired pro-islet amyloid polypeptide processing but due to impaired insulin maturation. Proinsulin crosses the blood-brain-barrier but does not induce central satiety. Accordingly, insulin therapy prevents hyperphagia. Further, islet PC1/3 expression levels negatively correlate with body mass index in humans. In this work, we show that impaired PC1/3-mediated proinsulin processing, as observed in human prediabetes, promotes hyperphagic obesity.

## Abstract (web summary)

Defective insulin secretion is observed early in the development of diabetes. Here the authors report that β cell-specific deficiency of the insulin prohormone convertase 1/3 (PC1/3) leads not only to hyperglycemia, but also to hyperphagic obesity in mice.

## Introduction

Proprotein convertase subtilisin/kexin type 1 (*PCSK1*, PC1/3) is a protease expressed in neuroendocrine tissues. PC1/3 processes and thus activates various central propeptides that are involved in body weight regulation including proopiomelanocortin (POMC), pro-neuropeptide Y (NPY), and pro-Agouti-related protein (AgRP) as well as peripheral satiety hormones, including proghrelin, pro-cholecystokinin (CCK), and proglucagon (yielding glucagon-like peptide 1). Thus, it is not surprising that loss-of-function mutations in *PCSK1* in humans lead to a complex set of endocrinopathies including malabsorptive diarrhea, hypogonadotropic hypogonadism, adrenal malfunction, central diabetes insipidus, and hyperphagic obesity^1–5^. Furthermore, linkage positional cloning, candidate gene, and genome-wide association studies identified common nonsynonymous polymorphisms in *PCSK1* to confer risk of obesity^6,7^. *Pcsk1* null mice are runted and not obese and therefore do not mimic the human phenotype^8^. However, mice that express a truncated PC1/3 variant develop hyperphagic obesity and increased metabolic efficiency^9^. POMC deficiency and the resulting lack of POMC-derived peptides is associated with severe obesity in humans and rodents^10,11^ and hence impaired POMC processing might mediate hyperphagia in individuals with PC1/3 deficiency.

Alternatively, we hypothesized that lack of proinsulin processing to insulin by PC1/3 in pancreatic β cells may be implicated in obesity. Indeed, the insulin receptor is widely expressed in the brain^12^ and administration of insulin directly to the brain reduces food intake and body weight gain in baboons and rodents^13,14^. In contrast, inhibition of central insulin signaling increases food intake and body weight gain^15^. In humans, intranasal insulin application induces satiety and reduces body weight gain^16,17^. Furthermore, brain-specific insulin receptor ablation increases food intake in rodents^18,19^. Moreover, obesity is associated with central insulin resistance^20^ or reduced insulin transport into the brain^21^. Since less than 1% of peripheral insulin is taken up by the brain^22^ via a saturable receptor-mediated process^23,24^ and proinsulin has a 100-fold reduced affinity for the insulin receptor^25^, it is conceivable that impaired proinsulin processing could lead to reduced central insulin action and thus hyperphagic obesity.

Here, we developed genetic models allowing spatiotemporal ablation of *Pcsk1* in mice. We show that central PC1/3 deficiency leads to endocrinopathies, but only mildly affects body weight regulation. In contrast, pancreatic β cell-specific *Pcsk1* ablation leads to profound hyperphagic obesity mediated by the lack of mature insulin. Furthermore, *PCSK1* expression in human islets negatively correlated with body mass index. Our results suggest that PC1/3 deficiency leads to obesity due to the absence of insulin-targeted anorexic pathways.

## Results

### Induced whole-body PC1/3 deletion leads to pronounced hyperphagic obesity

**Fig. 1:**
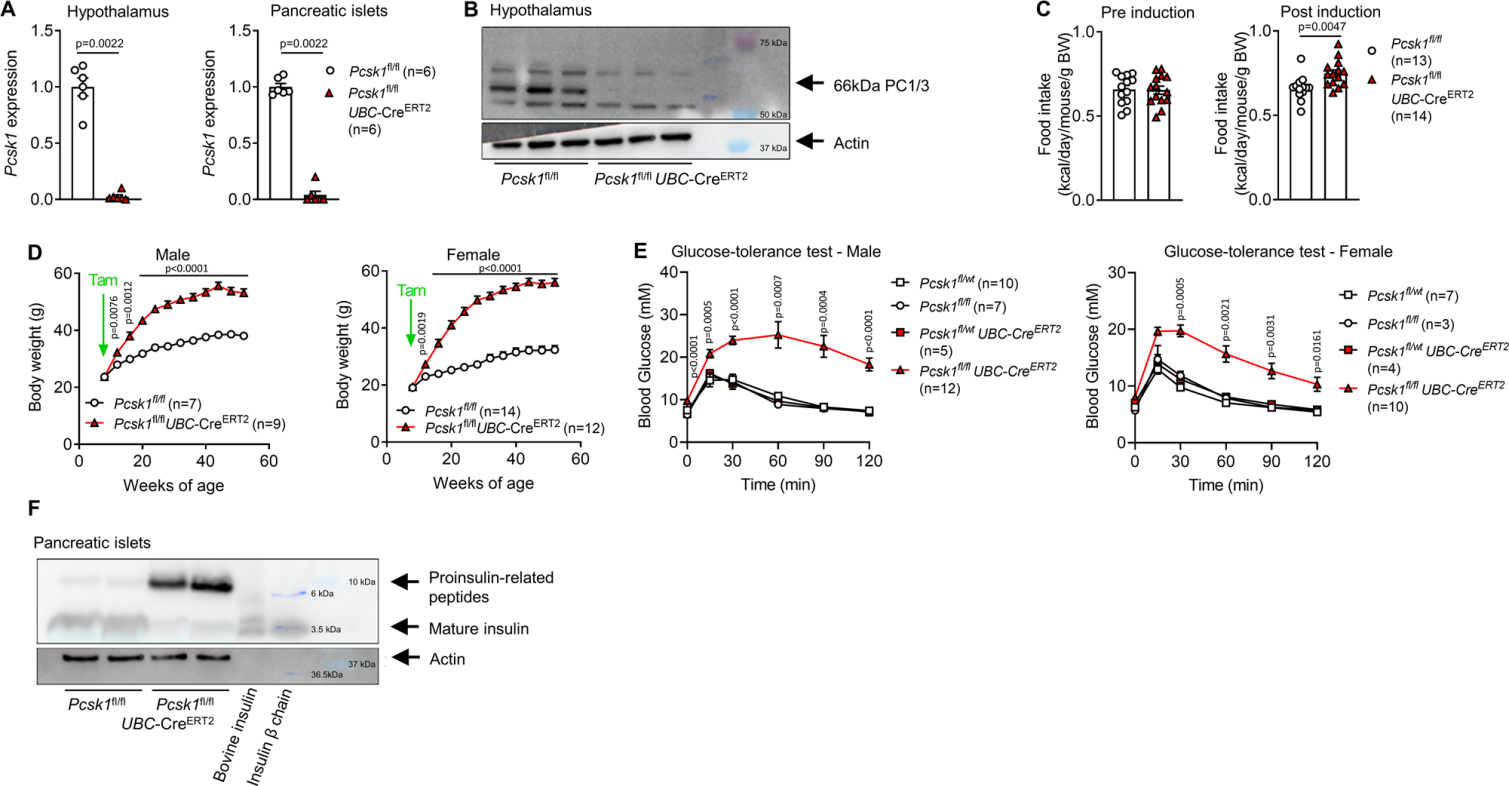
Induced whole-body PC1/3 deletion leads to pronounced hyperphagic obesity. **A** Normalized *Pcsk1* expression in the hypothalamus and pancreatic islets isolated from 12-week-old mice 4 weeks after knockout induction. **B** PC1/3 protein expression in hypothalami isolated from 14-week-old mice 5 weeks after knockout induction. Note: signal at 55 kDa is unrelated to PC1/3 and known to appear in this batch of antibody. **C** Food intake pre (from 7 to 8 weeks of age) and post (10–11 weeks of age) induction at 8 weeks of age. Each dot represents the average of a cage with 2–3 mice. **D** Body weight development. **E** Circulating glucose concentration after injection of 2 g kg^−1^ glucose at 12 weeks of age 4 weeks post induction. Indicated *p* values relate to the comparison of *Pcsk1*^fl/fl^ vs *Pcsk1*^fl/fl^ *UBC*-Cre^ERT2^. **F** Proinsulin processing assessed by western blotting in pancreatic islets isolated from 12-week-old mice 4 weeks after induction. Open squares: *Pcsk1*^fl/wt^, open circles: *Pcsk1*^fl/fl^, red squares: *Pcsk1*^fl/wt^ *UBC*-Cre^ERT2^, red triangles: *Pcsk1*^fl/fl^ *UBC*-Cre^ERT2^. Data are presented as mean values ± SEM. **A** and **C** were analyzed by a two-sided Mann–Whitney *U* test and **D** and **E** by a mixed-effects analysis with Holm–Sidak’s multiple comparisons post hoc test. Source data are provided as a Source Data file.

Human PC1/3 deficiency leads to severe obesity while *Pcsk1* null mice are runted and have a high mortality rate due to developmental defects^8^. Consistent with these data, mice on a C57BL6/N genetic background carrying a knockout-first conditional-ready allele that disrupts *Pcsk1* did not produce homozygous knockout mice when intercrossed (34 pups from 3 different breeding pairs, expected: 8 homozygous mice, obtained: 0). To circumvent embryonic lethality, we excised the FRT-flanked cassette and generated tamoxifen-inducible whole-body PC1/3 knockout mice (*Pcsk1*^fl/fl^ *UBC*-Cre^ERT2^). Four weeks after induction, *Pcsk1* expression in the hypothalamus and pancreatic islets was efficiently ablated, and PC1/3 protein was absent in the hypothalamus (Fig. 1A, B). In agreement with the clinical presentation of PC1/3 deficiency in humans, food intake increased by 16.9 ± 6.7% and mice of both sexes rapidly became obese (Fig. 1C, D). Glucose tolerance was impaired, likely due to a lack of mature insulin (Fig. 1E, F). Thus, *Pcsk1* ablation in early adulthood in rodents phenocopies hyperphagic obesity as seen in human PC1/3-obesity^3,4^.

### *POMC*-specific PC1/3 ablation induces mild and transient overweight

**Fig. 2:**
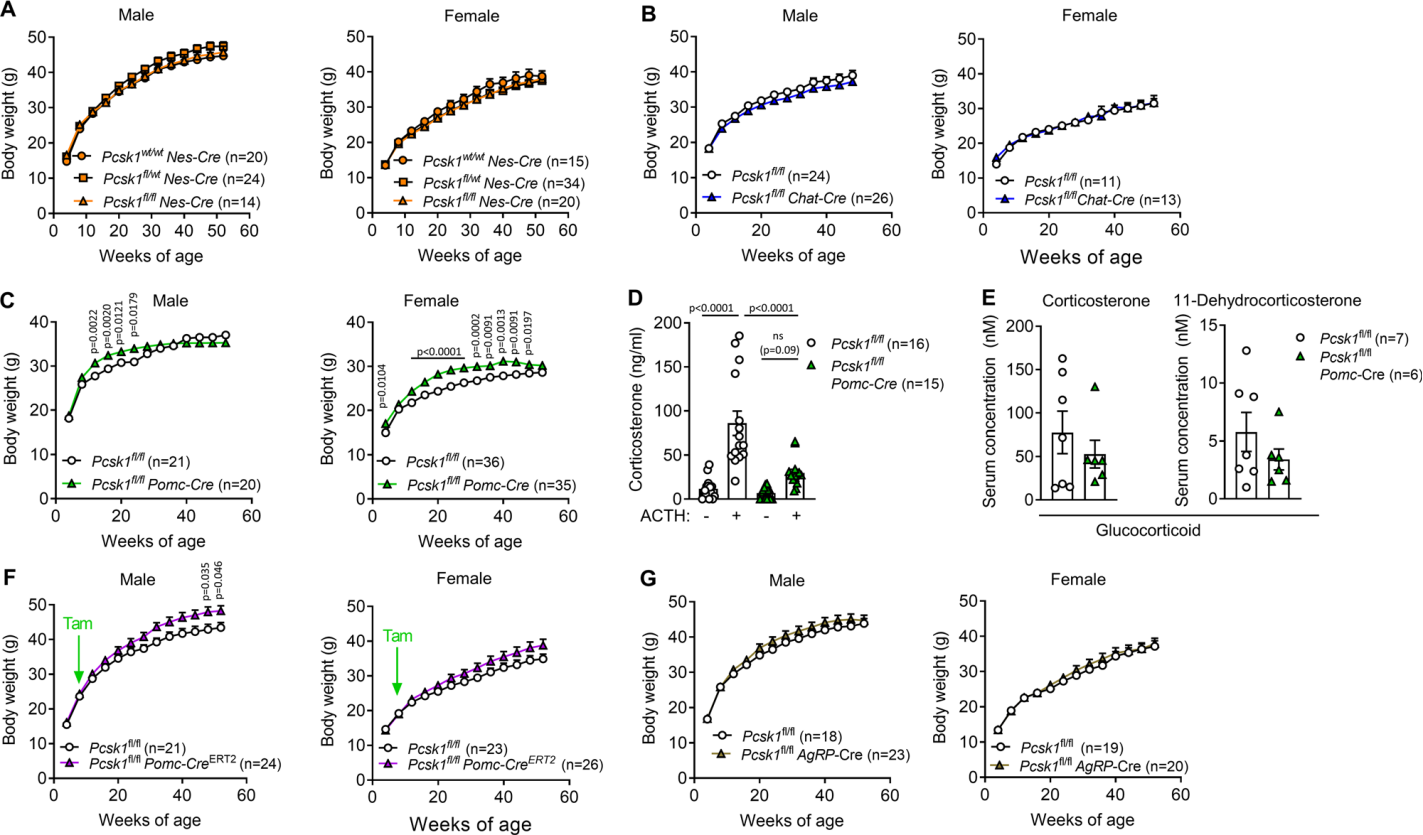
POMC-specific PC1/3 ablation induces mild and transient overweight. **A** Body weight development in *Pcsk1*^fl/fl^ *Nes*-Cre and control littermate mice. **B** Body weight development in *Pcsk1*^fl/fl^ *Chat*-Cre and control littermate mice. **C** Body weight development in *Pcsk1*^fl/fl^ *Pomc*-Cre and control littermate mice. **D** Circulating corticosterone concentrations before and 1 h after injection of 10 µg kg^−1^ human ACTH at 9:00 in female *Pcsk1*^fl/fl^ *Pomc*-Cre mice. **E** Circulating steroid concentrations at 11:00 in 12-week-old male *Pcsk1*^fl/fl^ *Pomc*-Cre mice. **F** Body weight development in *Pcsk1*^fl/fl^ *Pomc*-Cre^ERT2^ and control littermate mice. **G** Body weight development in *Pcsk1*^fl/fl^ *AgRP*-Cre and control littermate mice. Orange circles: *Pcsk1*^wt/wt^ *Nes*-Cre, orange squares: *Pcsk1*^fl/wt^ *Nes*-Cre, open circles: *Pcsk1*^fl/fl^, colored triangles: knockouts (orange: *Nes*-Cre, blue: *Chat*-Cre, green: *Pomc*-Cre, purple: *Pomc*-Cre^ERT2^, golden: *AgRP*-Cre). Data are presented as mean values ± SEM. **A**, **B**, **C**, **F**, and **G** were analyzed by a mixed-effects analysis with Holm–Sidak’s multiple comparisons post hoc test. **D** was analyzed by a one-way ANOVA with Holm–Sidak’s multiple comparisons post hoc test. **E** was analyzed by a two-sided Mann–Whitney *U* test. Source data are provided as a Source Data file.

To test whether the lack of processed central satiety hormones mediates the obesity associated with PC1/3 deficiency, we broadly ablated PC1/3 in the brain. Since *Nes*-Cre^1Kln^ driver mice show reduced body weight compared to wild-type controls, we generated littermate mice that all express Cre. Surprisingly, neither heterozygous *Pcsk1*^fl/wt^ *Nes*-Cre nor homozygous *Pcsk1*^fl/fl^ *Nes*-Cre mice of either sex showed altered body weight development (Fig. 2A). Challenging these mice with a high-fat diet also did not change body weight development or glycemia between genotypes (Supplementary Fig. S1A, B). Similarly, *Pcsk1*^fl/fl^ C*hat*-Cre of both sexes had similar body weights compared to controls (Fig. 2B). Within the central nervous system, PC1/3 processes and thereby activates several anorexigenic propeptides including POMC to pro-adrenocorticotropic hormone (proACTH). POMC ablation is associated with obesity in humans and rodents^10,11^, highlighting impaired POMC processing as a prime potential mediator of PC1/3-related obesity. Compared to controls, *Pcsk1*^fl/fl^ *Pomc*-Cre mice showed mild overweight which normalized at around 6 and 12 months of age in males and females, respectively (Fig. 2C). Western blotting of pituitary extracts showed that *Pcsk1*^fl/fl^ *Pomc*-Cre mice can process POMC to proACTH (Supplementary Fig. S1C), suggesting that PC1/3 is not essential to process POMC. However, ACTH-stimulation testing revealed that *Pcsk1*^fl/fl^ *Pomc*-Cre mice suffer from adrenal insufficiency (Fig. 2D). Supporting this observation, serum glucocorticoid levels (corticosterone and 11-dehydrocorticosterone, corresponding to cortisol and cortisone in human, respectively) were moderately reduced in *Pcsk1*^fl/fl^ *Pomc*-Cre mice while mineralocorticoids (aldosterone, 11-deoxycorticosterone and progesterone) did not differ from control mice (Fig. 2E and Supplementary Fig. S1D). The androgens testosterone and androstenedione were reduced in *Pcsk1*^fl/fl^ *Pomc*-Cre mice, suggesting decreased gonadal steroidogenesis (Supplementary Fig. S1E). To check whether the unexpectedly mild gain in body weight in *Pomc*-specific PC1/3 knockout mice was due to compensatory mechanisms during development, we next deleted PC1/3 in POMC-expressing cells in adulthood. Male mice developed mild late-onset obesity, while this did not reach statistical significance in females (Fig. 2F).

The orexigenic hormone AgRP is processed by PC1/3^26^ and ablation of AgRP itself or AgRP/NPY neurons in adult mice leads to cessation of feeding^27–29^. However, *Pcsk1*^fl/fl^ *AgRP*-Cre mice had a normal body weight development (Fig. 2G). Importantly, Cre-only control mice of all Cre driver lines used so far did not show a body weight phenotype (Supplementary Fig. S1F–J). Taken together, these data suggest that in rodents whole-body but not neuronal ablation of PC1/3 leads to profoundly increased body weight development. PC1/3 deficiency-related obesity must therefore be mediated by other, potentially peripheral, PC1/3 targets.

### β cell-specific *Pcsk1* ablation blocks proinsulin processing and induces hyperglycemia but not overt diabetes

**Fig. 3:**
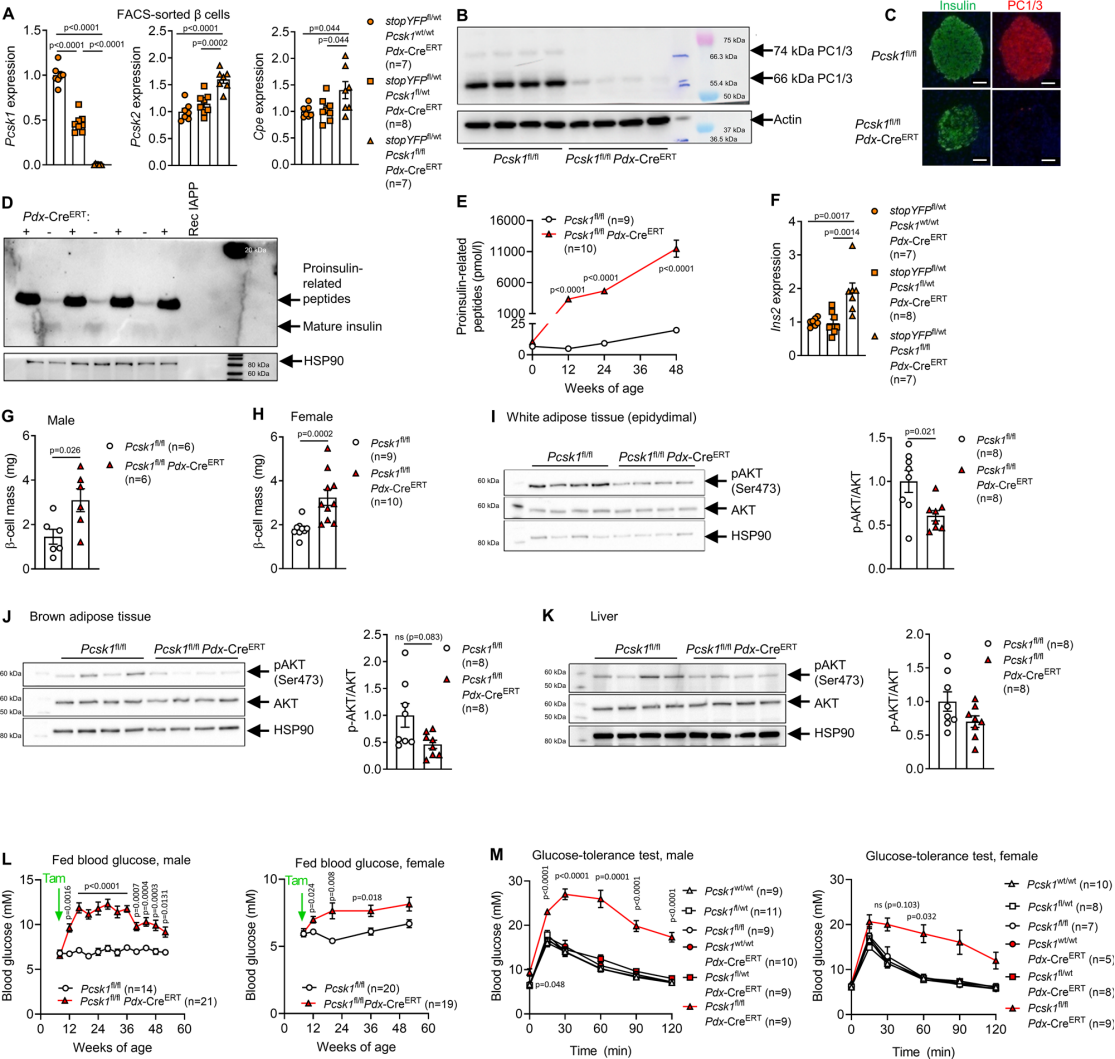
β cell-specific Pcsk1 ablation blocks proinsulin processing and induces hyperglycemia but not overt diabetes. **A** RNA analysis of FACS-sorted β cells (YFP+) dispersed from islet isolated from 12-week-old mice 4 weeks after induction. **B** PC1/3 western blot of isolated islets 15 weeks after induction. **C** Insulin and PC1/3-stained pancreatic section. Scale bar, 50 μm. **D** Insulin western blot of whole islets isolated from 12-week-old mice 4 weeks after induction. **E** Circulating levels of proinsulin-related peptides in male mice. **F** RNA analysis of FACS-sorted beta cells (YFP+) dispersed from islet isolated from 12-week-old mice 4 weeks after induction. **G**, **H** β-cell mass in 20-week-old mice 12 weeks after induction. **I**–**K** AKT western blot in white adipose tissue, brown adipose tissue, and liver after injection of 2 g kg^−1^ glucose following an overnight fast in male mice 4 weeks after induction. **L** Glycemia of fed mice (8 h after lights on). **M** Circulating glucose concentration after injection of 2 g kg^−1^ glucose at 12 weeks of age 4 weeks after induction. Orange circles: stop*YFP*^fl/wt^ *Pcsk1*^wt/wt^ *Pdx*-Cre^ERT^, orange squares: stop*YFP*^fl/wt^ *Pcsk1*^fl/wt^ *Pdx*-Cre^ERT^, orange triangles: stop*YFP*^fl/wt^ *Pcsk1*^fl/fl^ *Pdx*-Cre^ERT^, open triangles: *Pcsk1*^wt/wt^, open squares: *Pcsk1*^fl/wt^, open circles: *Pcsk1*^fl/fl^, red circles: *Pcsk1*^wt/wt^ *Pdx*-Cre^ERT^, red squares: *Pcsk1*^fl/wt^ *Pdx*-Cre^ERT^, red triangles: *Pcsk1*^fl/fl^ *Pdx*-Cre^ERT^. Data are presented as mean values ± SEM. **A** and **F** were analyzed by a one-way ANOVA with Holm–Sidak’s multiple comparisons post hoc test. **E**, **L**, and **M** were analyzed by a mixed-effects analysis with Holm–Sidak’s multiple comparisons post hoc test. **G**–**K** were analyzed by a two-sided Mann–Whitney *U* test. Source data are provided as a Source Data file.

One cell type that could mediate the obesity related to PC1/3 deficiency is the pancreatic β cell. Islet β cells secrete insulin and islet amyloid polypeptide (IAPP), both of which are dependent on PC1/3 processing and are regulators of satiety^13,30^. Therefore, we generated *Pcsk1*^fl/fl^ *Pdx*-Cre^ERT^ mice. Nearly all (98%) β cells expressed Cre recombinase-mediated reporter fluorescence (Supplementary Fig. S2A). *Pcsk1* gene expression was strongly reduced while *Pcsk2* and *Cpe* (encoding the insulin processing proteases PC2 and CPE, respectively) were upregulated and the prohormone convertase inhibitors *Pcsk1N* and *Scg5* were unchanged in fluorescence-activated cell sorting (FACS)-sorted β cells (Fig. 3A and Supplementary Fig. S2B). PC1/3 protein was efficiently ablated in isolated pancreatic islets and pancreatic sections (Fig. 3B, C). This effect was long-lasting, as islet *Pcsk1* was still absent 75 weeks post knockout induction (Supplementary Fig. S2C). During development, *Pdx* is expressed in the pancreas, in certain brain regions, as well as in the gastrointestinal L cells (where PC1/3 activates the satiety hormone GLP-1) but is limited to the β cell and acinar cells in adulthood^31–33^. We, therefore, induced mice at 8 weeks of age. Circulating concentrations of active GLP-1, intestinal GLP-1 content, as well as *Pcsk1* gene expression in the hypothalamus, pituitary, olfactory bulb, amygdala, and adrenal gland were unchanged in *Pcsk1*^fl/fl^ *Pdx*-Cre^ERT^ mice (Supplementary Fig. S2D–F). These data demonstrate that *Pcsk1*^fl/fl^ *Pdx*-Cre^ERT^ mice are an efficient model for β cell-specific *Pcsk1* ablation. *Pcsk1*^fl/fl^ *Pdx*-Cre^ERT^ mice were unable to produce mature insulin, whereas proinsulin-related peptides were markedly increased in the islets as well as in the circulation, and further increased over time, likely to compensate for the lack of mature insulin (Fig. 3D, E). Insulin gene expression, islet number, and size as well as pancreas weight were increased, resulting in doubled β-cell mass (Fig. 3F–H and Supplementary Fig. S2G–I). Furthermore, β cells had a degranulated appearance, suggesting that they were metabolically stressed (Supplementary Fig. S2J). Protein kinase B (AKT) was still phosphorylated in response to glucose in fat and liver, albeit to a lesser extent than in control mice, likely due to immature insulin’s bioactivity^25^ (Fig. 3I–K). Mice of both sexes were hyperglycemic but the survival frequency remained comparable to control littermates (Fig. 3L). Glucose tolerance was impaired and further deteriorated with increased age and high-fat diet feeding, yet mice of both sexes did not become catabolic (Fig. 3M and Supplementary Fig. S2K, L). *Pcsk1*^fl/fl^ *Pdx*-Cre^ERT^ mice prior to induction, heterozygous knockout mice, and Cre controls did not show glucose intolerance (Fig. 3M and Supplementary Fig. S2M). These data show that *Pcsk1*^fl/fl^ *Pdx*-Cre^ERT^ mice exhibit features of diabetes but counteract hyperglycemia by increasing the production and secretion of insulin precursors.

### Deficiency in proinsulin processing mediates hyperphagic obesity

**Fig. 4:**
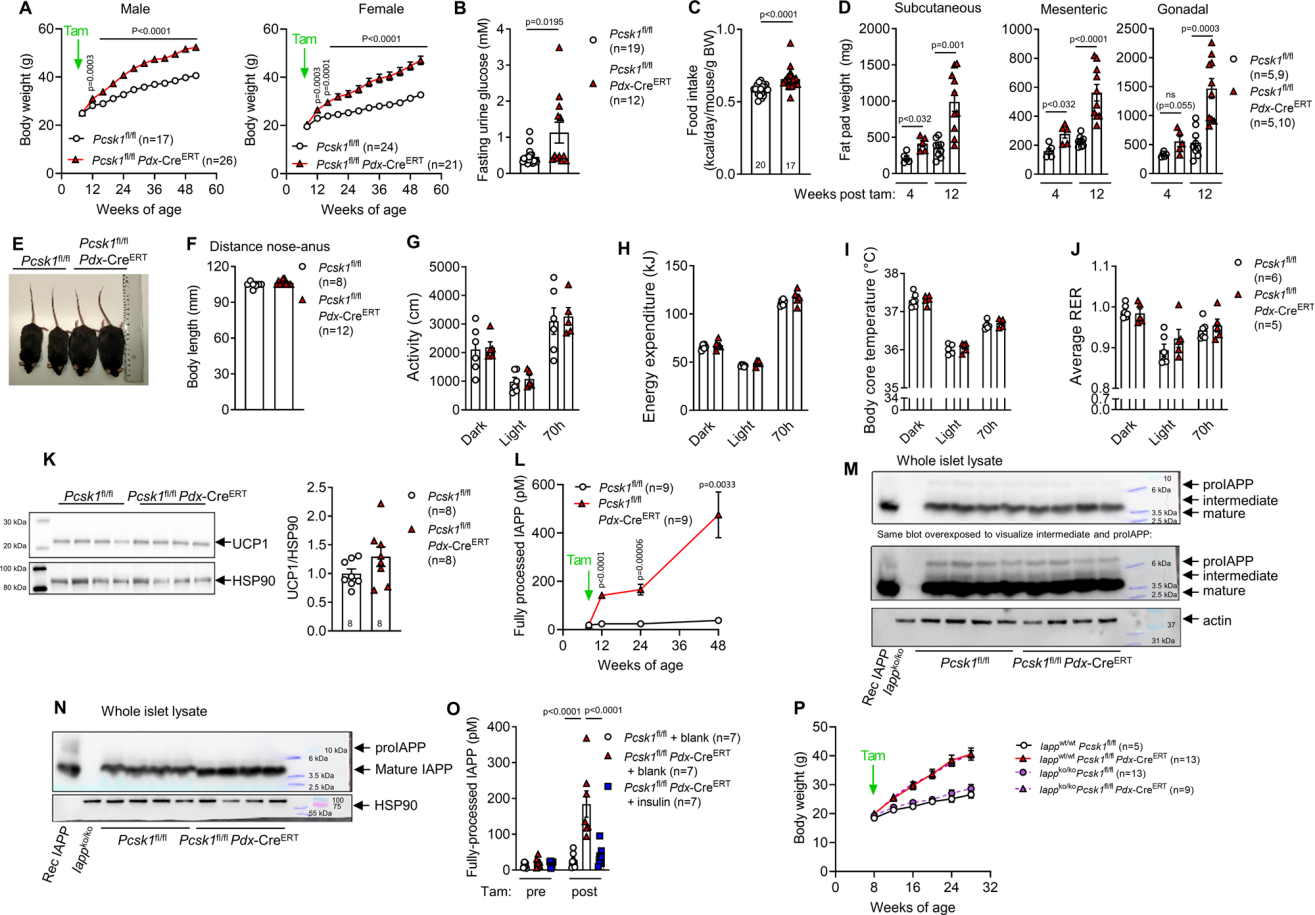
Deficiency in proinsulin processing mediates hyperphagic obesity. **A** Body weight development. **B** Glucose concentration in urine after a 6-h-fast 12 weeks post knockout induction. **C** Food intake 2 weeks (from 10 to 11 weeks of age) post induction at 8 weeks of age (each dot represents the average of 2–3 mice per cage). **D** Fat pad weights isolated 4 or 12 weeks post induction from female mice (littermates on both timepoints). **E** Picture of 49-week-old males induced at 8 weeks of age. **F** Distance snout to anus in 76–80-week-old male mice induced at 8 weeks of age. **G**–**J** Physical activity (**G**), energy expenditure (**H**), body core temperature (**I**), and respiratory exchange ratio (**J**) at 12 weeks of age 4 weeks post induction. **K** Western blot of brown adipose tissue after injection of 2 g kg^−1^ glucose following an overnight fast in mice 4 weeks after induction. **L** Circulating fully processed IAPP. **M**, **N** Islet IAPP protein probing with an antibody that detects all species of IAPP (**M**) or only fully processed (amidated) IAPP (**N**). **O** Circulating IAPP pre (8 weeks of age) and 3 weeks post induction in mice transplanted with insulin pellets or blanks. **P** Body weight development in triple-transgenic female littermate mice. Open circles: *Pcsk1*^fl/fl^, red triangles: *Pcsk1*^fl/fl^ *Pdx*-Cre^ERT^, blue squares: *Pcsk1*^fl/fl^ *Pdx*-Cre^ERT^ with insulin pellet implants, purple circle: *Iapp*^ko/ko^ *Pcsk1*^fl/fl^, purple triangle: *Iapp*^ko/ko^ *Pcsk1*^fl/fl^ *Pdx*-Cre^ERT^. Data are presented as mean values ± SEM. **A**, **L**, and **P** were analyzed by a mixed-effects analysis with Holm–Sidak’s multiple comparisons post hoc test. **B**–**D** and **F**–**K** were analyzed by a two-sided Mann–Whitney *U* test. **O** was analyzed by a one-way ANOVA with Holm–Sidak’s multiple comparisons post hoc test. Source data are provided as a Source Data file.

Similar to the inducible whole-body PC1/3 knockout mice (Fig. 1D), *Pcsk1*^fl/fl^ *Pdx*-Cre^ERT^ mice of both sexes showed a marked increase in body weight following knockout induction, despite a significant loss of calories due to hyperglycemia with subsequent glucosuria (Fig. 4A, B). Uninduced mice and Cre-only controls did not show altered body weight development (Supplementary Fig. S3A, B). The increase in body weight was driven by a 12% enhanced caloric intake (Fig. 4C and Supplementary Fig. S3C, D) and led to gradually enlarged fat pads, but not to a generally increased body size (Fig. 4D–F). Physical activity, energy expenditure, body core temperature, respiratory exchange ratio, and UCP1 expression in brown adipose tissue were not changed (Fig. 4G–K and Supplementary Fig. S3E–H), suggesting that energy output is not affected by β cell-specific ablation of PC1/3. Next, we investigated whether processing deficits in IAPP mediate the observed obesity phenotype. Surprisingly, *Pcsk1*^fl/fl^ *Pdx*-Cre^ERT^ mice showed circulating levels of fully processed IAPP and the concentration increased steadily with age (Fig. 4L). Western blotting with antibodies that react to all species of IAPP (Fig. 4M) or only to fully processed IAPP (Fig. 4N) confirmed that *Pcsk1*^fl/fl^ *Pdx*-Cre^ERT^ mice process proIAPP in the absence of β cell PC1/3. IAPP and insulin are co-secreted from β cells^34^ and normalizing proinsulin secretion by implanting *Pcsk1*^fl/fl^ *Pdx*-Cre^ERT^ mice with subcutaneous insulin pellets prevented the increase in circulating IAPP (Fig. 4O), suggesting that the increase in circulating IAPP is secondary to increased proinsulin secretion. Exogenous recombinant IAPP reduced food intake in control but not in *Pcsk1*^fl/fl^ *Pdx*-Cre^ERT^ mice (Supplementary Fig. S3I), suggesting that the constant exposure to high circulating levels of IAPP might induce resistance to central IAPP action. To test this hypothesis, we made *Pcsk1*^fl/fl^ *Pdx*-Cre^ERT^ mice on an *Iapp* null background and found that the body weight phenotype does not depend on the presence of IAPP (Fig. 4P). IAPP also slows gastric emptying but despite high peripheral levels of IAPP, the rate of gastric emptying was unchanged (Supplementary Fig. S3J). Furthermore, circulating satiety hormones derived from other organs (FGF21, ghrelin, leptin, PYY) were unchanged (Supplementary Fig. S3K). Finally, ghrelin expression in the stomach of *Pcsk1*^fl/fl^ *Pdx*-Cre^ERT^ mice remained unchanged (Supplementary Fig. S3L). These results indicate that obesity in β cell-specific PC1/3 knockout mice is mediated by increased food intake and that this effect is independent of IAPP.

### Incompletely processed proinsulin crosses the blood–brain barrier but does not induce central satiety

**Fig. 5:**
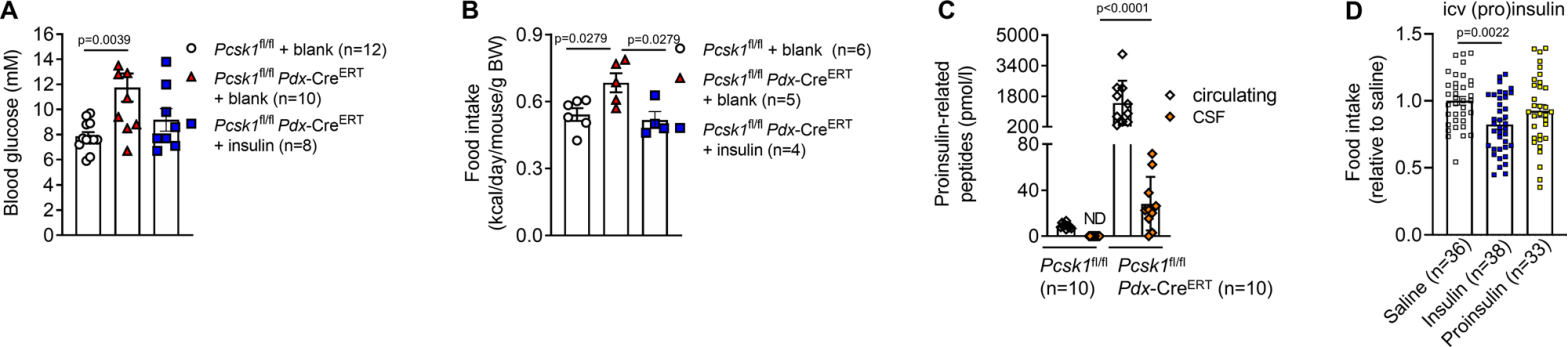
Incompletely processed proinsulin crosses the blood–brain barrier but does not induce central satiety. **A**, **B** Blood glucose concentrations (**A**) and food intake (average of 2 mice per cage) (**B**) 2 weeks (10–11 weeks of age) post induction in mice transplanted with insulin pellets or blanks 1 week after the first tamoxifen dose. **C** Circulating and cerebrospinal fluid levels of proinsulin-related peptides (isolated from the same mouse) in mice at various ages. **D** 20 h cumulative food intake in female C57BL/6N mice following an intracerebroventricular injection of saline, 0.4 µU insulin, or an equimolar amount of proinsulin. Open circles: *Pcsk1*^fl/fl^, red triangles: *Pcsk1*^fl/fl^ *Pdx*-Cre^ERT^, blue squares: *Pcsk1*^fl/fl^ *Pdx*-Cre^ERT^ with insulin pellet implants, open diamonds: circulating, orange diamonds: cerebrospinal fluid, open squares: icv saline, blue squares: icv insulin, yellow squares: icv proinsulin. Data are presented as mean values ± SEM. **A**–**D** were analyzed by a one-way ANOVA with Holm–Sidak’s multiple comparisons post hoc test. Source data are provided as a Source Data file.

Next, we investigated whether impaired proinsulin processing may mechanistically underlie hyperphagic obesity. *Pcsk1*^fl/fl^ *Pdx*-Cre^ERT^ were implanted subcutaneously with insulin pellets which prevented hyperglycemia and hyperphagia (Fig. 5A, B). Since insulin crosses the blood–brain barrier in a receptor-mediated manner^23,24^ and proinsulin has a low affinity for the insulin receptor^25^, we hypothesized that proinsulin is unable to reach the brain and induce central signaling. Therefore, we assessed plasma and cerebrospinal fluid (csf) and found proinsulin-related peptide levels to be undetectable in csf from control mice but readily measurable in csf from *Pcsk1*^fl/fl^ *Pdx*-Cre^ERT^ mice, on average corresponding to 2.5% of circulating levels (Fig. 5C). Intracerebroventricular injection of insulin into the third ventricle reduced food intake but this was not observed for an equimolar dose of proinsulin (Fig. 5D). These data imply that immature insulin can cross the blood–brain barrier but is unable to induce central satiety.

### Islet *PCSK1* expression correlates with body mass index in humans

**Fig. 6:**
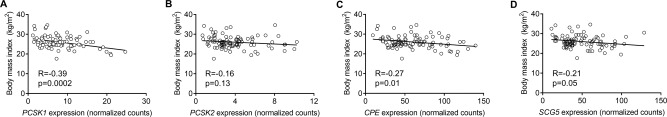
Islet PCSK1 expression negatively correlates with body mass index in humans. **A**–**D** Correlation of body mass index of donors and whole islet mRNA expression of **A** *PCSK1*, **B** *PCSK2*, **C** *CPE*, and **D** *SCG5*. The data set did not contain *PCSK1N*. Data extracted from GSE50244. *n* = 89. Source data are provided as a Source Data file.

In humans, inactivation mutations of PC1/3 lead to severe obesity^3^. However, the contribution of tissue-specific *PCSK1* expression is unknown. Therefore, we next assessed RNA expression levels in a published RNA-seq data set of human islets^35^. Body mass index negatively correlated with *PCSK1* and *CPE* expression but not with *PCSK2* or *SCG5*, suggesting that islet PC1/3 could be an important determinant of body weight regulation in humans (Fig. 6A–C).

## Discussion

Our work shows that impaired proinsulin processing is an important contributor to PC1/3-related hyperphagic obesity. While central PC1/3 deficiency only mildly and transiently increased body weight development, β cell-specific ablation of PC1/3 induced pronounced obesity in our preclinical mouse models and insulin therapy prevented hyperphagia.

Peripheral signals that influence energy intake are processed by central circuits and in this process, hypothalamic POMC neurons are a major integration site. Activation of POMC suppresses appetite^36^, while POMC inhibition and mutations that reduce POMC activity induce obesity in rodents, dogs, and humans^10,11,36,37^. Since PC1/3 cleaves POMC to the anorectic α-MSH, the canonical view is that PC1/3-related obesity could be regulated by reduced processing of POMC. However, there seems to be a disconnect between POMC processing and body weight development. Hypothalamic extracts isolated from the obese *Pcsk1* N222D mutant mouse still have 55% α-MSH content^9^ but PC2 null mice who produce no α-MSH^38^ are not obese^39^. Furthermore, pituitaries isolated from whole-body PC1/3 knockout mice still have 50% of α-MSH content^8^ and ablation of *PCSK1* in human embryonic stem cell-derived hypothalamic neurons had no effect on α-MSH content^40^. Thus, PC1/3 is dispensable for POMC processing and the latter does not necessarily regulate body weight development. Supporting this notion, deletion of PC1/3 specifically in POMC neurons in adulthood did not change body weight development for up to 9 weeks after knockout induction^41^. We confirmed these findings but in addition observed mild late-onset obesity in aged mice. Furthermore, we found life-long *Pcsk1* knockout in POMC neurons to induce obesity which resolved after 6 and 12 months of age in male and female mice, respectively. These mice had detectable levels of ACTH but an insufficient amount for normal adrenal function. Gonadal androgens were reduced in these mice but the breeding performance was normal. Similarly, humans with PC1/3 deficiency suffer from hypogonadism^1,3^ and our data suggests that this is partly mediated by defective PC1/3 activity in POMC neurons.

There are considerable phenotypic differences in the presentation of PC1/3 deficiency between humans and rodents. *Pcsk1* null mice are runted or suffer from preimplantation lethality^8,42^ and a *Cmv*-Cre version of our mouse model is also not viable. This apparent contrast to patients with PC1/3 deficiency could arise from the fact that levels of adrenal androgens are high during early development in humans but mice solely depend on gonadal androgen synthesis. Thus, mice might display a more severe pathology, as a lack of gonadal androgens in humans may be partly compensated for.

In contrast to our whole-body PC1/3 knockout model, broad or targeted central knockout lines unexpectedly had only mild transient body weight phenotypes. Therefore, we reasoned that there might be an important, potentially peripheral, PC1/3 target that regulates body weight development. The pancreatic β cell seemed to be a logical candidate, as its major products, insulin and IAPP are processed by PC1/3 and both activate central satiety signaling pathways. Indeed, *Pcsk1*^fl/fl^ *Pdx*-Cre^ERT^ mice with knockout induction in adulthood quickly became hyperphagic and obese. Conceptually, these mice represent a model of severe type 2 diabetes, as they increase the production and secretion of less-active and immature insulin to keep glycemia under control. Caloric intake was elevated and despite the loss of calories due to glucosuria they gained body weight in excess. They phosphorylated AKT and did not develop life-threatening hyperglycemia, nor did they become catabolic even though these mice lacked fully processed insulin. In contrast, a lack of both mature and immature insulin, as observed in the context of type 1 diabetes, leads to severe catabolism. Noteworthy, defective islet prohormone processing and increased proinsulin levels were also described in individuals prior to and after the onset of type 1 diabetes (reviewed:^43^). However, it is currently unknown whether an islet intrinsic prohormone processing defect predisposes individuals to develop autoimmune diabetes or whether increased secretory demand due to autoimmune β-cell destruction leads to a relative increase in proinsulin to insulin ratio.

The investigation of the β cell-derived satiety hormone IAPP in *Pcsk1*^fl/fl^ *Pdx*-Cre^ERT^ mice yielded unexpected results. First, in contrast to proinsulin, proIAPP was processed in the absence of PC1/3, possibly by PC2. Indeed, β cell expression of PC2 and CPE, two other proteases involved in proinsulin/proIAPP processing, were increased. Second, circulating levels of IAPP were increased and this was prevented by insulin therapy, confirming that insulin and IAPP are co-secreted^34^. Despite very high circulating levels of endogenous IAPP, these mice were still obese, suggesting that IAPP did not induce satiety. Third, *Pcsk1 Iapp* double knockout mice had the same body weight phenotype as *Pcsk1* single knockout mice expressing *Iapp*. This shows that the obesity phenotype was not due to IAPP resistance induced by constant high circulating levels of IAPP. In this context, it is interesting that in contrast to proinsulin, proIAPP was not found to be elevated in individuals with type 2 diabetes^44–47^. These data prove that in our mouse model IAPP did not regulate obesity.

On the other hand, impaired proinsulin processing mediated the obesity phenotype in *Pcsk1*^fl/fl^ *Pdx*-Cre^ERT^ mice, and insulin replacement by implanting subcutaneous insulin pellets prevented hyperphagia. The anorectic effect of central insulin^13,14,16,17,48^ and the orexigenic effect of central insulin deficiency^15,18–20^ is well described (reviewed:^49^). Since insulin uptake into the brain^22^ is a saturable receptor-mediated process^23,24^, we hypothesized that *Pcsk1*^fl/fl^ *Pdx*-Cre^ERT^ mice become obese because incompletely processed insulin does not efficiently induce central satiety. Surprisingly, we readily detected proinsulin-related peptides in the csf of *Pcsk1*^fl/fl^ *Pdx*-Cre^ERT^ mice, suggesting that these peptides can cross the blood–brain barrier despite a 100-fold reduced affinity for the insulin receptor^25^. However, in support of our hypothesis, central injection of proinsulin did not induce satiety while injection of insulin did, despite the much longer half-life of proinsulin^50^. While a lack of central insulin action is the most straight-forward explanation for hyperphagia in these mice, we can currently not rule out that hyperproinsulinemia per se induces hyperphagia and that insulin therapy prevented this phenotype indirectly by reducing β-cell secretory activity and thus circulating proinsulin levels. An argument against this hypothesis is the observation that AKT phosphorylation in insulin target tissues was reduced and not increased in *Pcsk1*^fl/fl^ *Pdx*-Cre^ERT^ mice.

Although *Pdx* expression in adulthood is largely limited to β cells^32,33^, Cre recombination-mediated reporter gene expression in certain *Pdx*-Cre driver mice was also detected in the duodenum and in hypothalamic neurons^51,52^, raising the possibility that the observed hyperphagia could be mediated by non-β cell targets. Nevertheless, the levels of the intestinal PC1/3 target GLP-1 were not altered and we, as well as others^53^, have found the targeted gene not to be reduced in the hypothalamus when *Pdx*-Cre^ERT(Dam)^ driver mice were studied. Furthermore, none of the neuron-specific PC1/3 knockout models recapitulated the pronounced obese phenotype observed in whole-body or *Pcsk1*^fl/fl^ *Pdx*-Cre^ERT^ mice and insulin replacement therapy prevented hyperphagia. These data strongly suggest that *Pcsk1* deficiency in pancreatic β cells mediated the observed obesity phenotype. Noteworthy, both constitutive and adult-onset deletion of PC1/3 in *POMC*-expressing tissues induced obesity, although only mildly and transiently, showing that POMC processing regulates body weight development.

The complete absence of PC1/3 in humans^3–5,54^ and in our inducible whole-body knockout mouse model induces hyperphagic obesity. However, also a partial reduction in *Pcsk1* expression seems to regulate body weight development. Mouse models with reduced PC1/3 activity develop obesity^9,55^ and in human nonsynonymous polymorphisms in *PCSK1* confer risk of obesity^6,7^. Interestingly, prediabetes and type 2 diabetes, which are associated with obesity, are characterized by reduced *PCSK1* expression in pancreatic islets^35^ and impaired proinsulin processing, leading to an increased proinsulin to insulin ratio^45–47^. Insulin knockout mice are not viable^56^ but *Ins1* or *Ins2* null mice have normal circulating insulin levels and do not develop hyperglycemia or obesity^57^. Thus, reducing insulin dosage per se is not sufficient to alter body weight development. The increased insulin secretion observed in prediabetes may be the consequence of an increased insulin demand due to obesity-associated insulin resistance. Still, the existence of an intrinsic insulin secretion defect was proposed and is supported by GWAS studies^58,59^. Of particular interest are the above-mentioned polymorphisms in *PCSK1* that are associated with obesity^6,7^. It is conceivable that genetic predisposition to altered insulin expression and processing may promote obesity and not (only) be the consequence thereof^60^. Furthermore, Prader–Willi Syndrome, a genetic disease characterized by strong hyperphagia is associated with reduced levels of PC1/3 in islets^61^. Supporting a role for β cell-derived PC1/3 in human body weight regulation, we found body mass index to be negatively associated with islet *PCSK1* expression. Of note, the β cell endopeptidase *CPE* but not *PCSK2* also negatively correlated with body mass index. Interestingly, *Cpe* null mice are heavily obese and lack mature insulin^62^, while PC2 null mice are not obese^63^.

Taken together, our data reveal that the major mechanism for PC1/3 deficiency-associated obesity is not a defect in central processing and we suggest that the pancreatic β-cell-derived insulin is a major player in this pathology. Therefore, a judicious exogenous insulin therapy provided in a near-physiological dose may prevent obesity in affected individuals by inducing satiety without promoting exaggerated fat deposition.

## Methods

### Mice

All animal experiments were performed in accordance with the federal laws of Switzerland and approved by the cantonal veterinary office of Basel (approval numbers 2511 and 3045). Whole-body *Pcsk1* knockout mice were derived from embryonic stem cells carrying a transgene encoding an FRT-flanked lacZ and neo cassette with loxP sites around exon 5 of the *Pcsk1* gene (EUCOMM program) and these mice were subsequently crossed to FLP-transgenic mice^64^ to obtain *Pcsk1*^fl/fl^ mice^65^ (B6N-*Pcsk1*^tm1Boe^). *UBC*-Cre B6.Cg-*Ndor1*^Tg(UBC-cre/ERT2)1Ejb^ (Jax:007001^66^), B6.Cg-Tg (*Nes*-Cre)^1Kln^ (Jax:003771^67^), B6.FVB-Tg (*Pomc1*-Cre)^16Lowl^ (Jax: 010714^68^), B6.STOCK-*Agrp*^*tm1(cre)Lowl*^ (Jax:012899^69^), B6;129S6-*Chat*^*tm2(cre)Lowl*^ (Jax: 006410^70^) subsequently crossed to FLP-transgenic mice to excise the FRT-flanked cassette, B6.129(Cg)-*Gt(ROSA)26Sor*^*tm4(ACTB-tdTomato,-EGFP)Luo*^ (Jax: 007676^71^) and B6.129×1-*Gt(ROSA)26Sor*^*tm1(EYFP)Cos*^ (Jax: 006148^72^) were obtained in-house. B6N.STOCK-*Gt(ROSA)26Sor*^*tm1(FLP1)Dym* 64^ were obtained from Charles River (Sulzfeld, Germany). B6.STOCK Tg(*Pdx1*-Cre/Esr1*)^Dam^ (Jax: 024968^31^) were obtained from Michael B. Wheeler (University of Toronto). B6.129P2-*Iapp*^tm1Sgm^ ^73^ and B6.Cg-Tg(*Pomc*-Cre)^ERT2Jke^ ^74^ were obtained from Thomas A. Lutz (University of Zurich, Switzerland). Prior to intercrossing, all animals were fully backcrossed (at least 10 generations) onto a C57BL/6N (Charles River) genetic background. Mice were housed in groups of 2–6 mice in individually ventilated cages (Indulab, Gams, Switzerland) with standard bedding, nesting material, and a house, in two specific pathogen-free, temperature-controlled (21 ± 2 °C), humidity controlled (50–60%) facilities with a 12 h dark/12 h light cycle (lights on at 6:00), at the University of Basel. Mice received standard chow (3436, Kliba Nafag, Kaiseraugst, Switzerland) or lard-based 60 kJ% high-fat diet (E15742-34, ssniff Spezialdiäten GmbH, Soest, Germany) and water ad libitum. All studies were done using littermate controls. For inducible models, mice (including controls) received 125 mg kg^−1^ tamoxifen (T5648, Sigma-Aldrich, Buchs, Switzerland) dissolved in ethanol/corn oil (C8267, Sigma-Aldrich) on Monday, Wednesday, and Friday at 8 weeks of age by oral gavage. Mice of both sexes were used in most of the studies. Whenever possible, experimenters were blinded to the genotype of the experimental animal.

### Body weight development and food intake

Body weight was measured weekly using the dynamic weighing feature that averages measurements taken over a 5 s period (Scout STX, Ohaus, Nänikon, Switzerland). To increase readability, not all body weight data are depicted in the figures. Food intake was assessed by weekly measurement of the food given/left in the rack feeder of the cage.

### Glycemia and glucose tolerance

Blood glucose was measured with a glucometer (FreeStyle Freedom Lite, Abbott, Baar, Switzerland) from a drop of blood obtained from a small incision into the tail vein. Fasting blood glucose was obtained in animals deprived of food from 8:00 to 14:00. Blood was collected into heparinized capillaries by tail-tip bleeding and transferred to tubes containing EDTA, stored on ice, followed by centrifugation at 10,000 × g for 5 min at 4 °C and storage at −20 or −80 °C. Terminal blood was collected by cardiac puncture through the skin. Glucose tolerance was assessed by intra-peritoneal or oral application of 2 g kg^−1^ glucose after fasting from 8:00 to 14:00. Blood glucose was assessed prior to and 15, 30, 60, 90, and 120 min after glucose administration. Baseline blood glucose was measured once, all other timepoints twice. When the concentration of blood glucose was above the measurable range, 2 ul blood was mixed with 2 ul diluent 100 (R50AA-4, Meso Scale Diagnostics, Rockville, MD, USA) and measured by a glucometer. If the obtained values differed by more than 2 mM, a third measurement was obtained and the value furthest away was excluded. Initially, plasma insulin was assessed at 0, 15 and 30 min of the glucose tolerance test (K152BZC, Meso Scale Diagnostics) but this assay seemed to be highly cross-reactive with split proinsulins and therefore the data obtained from *UBC*-Cre^ERT2^ *Pcsk1*^fl/fl^ and *Pdx*-Cre^ERT^ *Pcsk1*^fl/fl^ could not be compared to their *Pcsk1*^fl/fl^ controls, making a useful interpretation obsolete.

### ACTH stimulation test

Mice were handled daily for at least 2 weeks to minimize stress responses. A blood sample was collected by tail-tip bleeding at 9:00, followed by intra-peritoneal injection of 10 μg kg^−1^ synthetic human ACTH (Synacthen). A second blood sample was collected 1 h later and plasma corticosterone was assessed (ADI-900-097, Enzo Life Sciences, Lausen, Switzerland).

### Gastric emptying test

Mice were deprived of food from 8:00 to 14:00, followed by oral gavage of 2 g kg^−1^ glucose and 100 mg kg^−1^ acetaminophen (A5000, Sigma-Aldrich). Plasma levels of acetaminophen were assessed using the Paracetamol Three Reagent System (K8002, Cambridge Life Sciences).

### RNA isolation and real-time quantitative PCR

Total RNA of various tissues was extracted using the NucleoSpin RNA Kit (740955, Macherey Nagel, Oensingen, Switzerland) and reverse transcribed using random hexamers and GoScript reverse transcriptase (A2801, Promega, Dübendorf, Switzerland). Quantitative real-time PCR was performed using GoTaq qPCR (A6002, Promega) and primer sets designed by Primer-BLAST (NCBI, Bethesda, MD, USA) manufactured by Microsynth (Balgach, Switzerland), run on an ABI 7500 Fast Real-Time PCR System (Thermo Fisher Scientific, Waltham, MA, USA). Whenever possible, primers were designed in an exon-exon spanning manner, and the melt curve, product size, and purity, as well as a serial dilution, were checked to ensure specificity. Primer sequences are listed in Supplementary Table S1. Gene expression was analyzed using the comparative 2^ΔΔCT^ method using actin, gapdh, and 18S as housekeeping genes.

### Protein isolation from tissue and western blotting

Tissues were dissected and dissolved in protein lysis buffer (20 mM Tris pH = 7.5, 1% Triton X-100, 150 mM NaCl, 10% glycerol, 1 mM Na3VO4, 10 mM NaF, 5 mM PMSF, 1 mM EDTA pH = 8.0) containing protease inhibitor cocktail (11836145001, Sigma-Aldrich), incubated for 15 min on ice, sonicated for 2 min (Elmasonic X-tra 150 H) and centrifuged for 10 min at 10,000 × g at 4 °C. The supernatant was stored at −80 °C. Protein content was assessed by a BCA assay (23225, Thermo Fisher Scientific). Then, 5–20 ug of the lysates were mixed with sample buffer (NP0007, Thermo Scientific Fisher) and sample reducing agent (NP0009, Thermo Scientific Fisher), loaded onto a gel (4–12% NP0336 or 12% NP034, Thermo Scientific Fisher) and separated and blotted onto a nitrocellulose membrane (9004700, BIO-RAD, Cressier, Switzerland) with the Invitrogen XCell Surelock Mini-Cell Electrophoresis System. The membrane was blocked in 5% milk in TBST, thereafter it was stained with the primary antibody at 4 °C overnight. On the next day, a secondary antibody coupled to horseradish peroxidase was added and incubated for 2 h at room temperature. SuperSignal™ West Pico PLUS Chemiluminescent Substrate was added and the membrane was imaged with the Fusion FX system (VILBER, France).

### Immunohistochemistry

Pancreata were dissected, weighed, and incubated in 4% buffered formalin (Formafix AG, Hittnau, Switzerland) overnight at 4 °C, thoroughly washed with PBS, processed on a TPC 15 DUO, and embedded in paraffin on a TES Valida (both: Medite, Dietikon, Switzerland) and sectioned into 5 μm slices on an HM340E microtome (Thermo Fisher Scientific). Tissue sections were deparaffinized, rehydrated, and incubated with antibodies raised against insulin, CD45 (to visualize lymph nodes), PC1/3, and DAPI (to visualize nuclei). Detailed information about the antibodies used is given in Supplementary Table S2. Slides were mounted using a fluorescence mounting medium (S3023 Agilent Dako, Basel, Switzerland) and analyzed.

### Islet isolation

Mice were euthanized and injected with collagenase (4189, Worthington, Lakewood, NJ, USA) through the bile duct. Pancreata were excised and incubated at 37 °C for 30 min with occasional shaking. The reaction was quenched by the addition of an HBSS medium containing 0.5 w/v BSA. The solution was filtered and islets resuspended in RPMI-1640 medium containing 11.1 mM glucose, 100 units ml^−1^ penicillin, 100 μg ml^−1^ streptomycin, 2 mM glutamax, 50 μg ml^−1^ gentamycin, 10 μg ml^−1^ fungizone and 10% FCS. Islets were hand-picked, lysed in RNA lysis buffer, and stored at −80 °C until RNA was isolated as described above.

### β-cell mass

Tissue sections from three to four different depths of the pancreas (at least 100 μm apart) were prepared as described above. Pancreas sections were imaged using an automated Eclipse Ni microscope (Nikon, Egg, Switzerland) with an Orca Flash 4.0 camera (Hamamatsu Photonics, Solothurn, Switzerland), a 4× Objective NA 0.2 (Nikon), and a PL-200 slide loading robot (PRIOR Scientific, Fulbourn, UK) using NIS Elements software v5. Ilastik software^75^ was trained to segment the pancreas area of stitched multichannel images, including channels for nuclei (DAPI), autofluorescence (GFP), lymph nodes (Alexa-555), and insulin (Alexa-647). The simple segmentation outputs of ilastik and corresponding original multichannel images were subsequently processed using Fiji^76^. In a first step, the ilastik simple segmentation recognition of the pancreas was checked and corrected manually if needed. In a second step, the β-cell identification on the corresponding channel was obtained by setting all pixels that do not overlap with the pancreas area to 0, thresholding using the Otsu method and performing a connected component analysis on objects bigger than 900 µm^2^ and having a circularity value bigger than 0.2. Insulin object recognition was adjusted manually if needed. All measurements (pancreas area and individual insulin areas) were saved to a spreadsheet. The Fiji analysis workflow was facilitated using a custom ImageJ script, which is available upon request from the corresponding author. The script was run on a remote-access server system (University of Basel). β-cell mass was calculated as pancreas weight (mg) × sum (insulin + area)/sum (pancreas area). All samples were handled in a blinded fashion by the same researcher.

### Flow cytometry and fluorescence-activated cell sorting (FACS)

Following pancreatic islet isolation, islets were washed and resuspended in 900 µl PBS + 0.5 mM EDTA. 200 µl digestion solution (0.01% trypsin + 0.5 mM EDTA) was added and incubated at 37 °C for 90 s with manual pipetting every 30 s. Nine milliliter cold FACS buffer (PBS + 0.5% BSA + 5 mM EDTA) was added to stop the digestion and the solution was centrifuged 350 × g for 5 min. The supernatant was removed and the cells were resuspended in 200 µl FACS buffer. Fc receptors were blocked by the addition of 2 µl anti-CD16/CD32 antibody and incubated for 15 min on ice. Immune cells were labeled by the addition of 2.5 µl CD45-APC antibody and incubation for 30 min. Cells were washed and resuspended in 100 ul FACS buffer and analyzed on a CytoFLEX flow cytometer (Beckman Coulter, Nyon, Switzerland) or a BD Fortessa (BD Biosciences, San Jose, USA) using FlowJo (v10.6.1, FlowJo LLC). If cells had to be sorted, a BD SORPAria 3, a BD FACSAria 3, or a BD Influx cell sorter (BD Biosciences) was used. See Supplementary Table S2 for more information about antibodies used.

### Hormone measurements

Proinsulin-related peptides were assessed by a proinsulin ELISA (10-1232-01, Mercodia, Uppsala, Sweden). According to the manufacturer’s instructions, this assay does not cross-react with mouse mature insulin or C-peptide but our data suggest that it also detects split proinsulins. IAPP was measured by ELISA (human mature IAPP, EZHA-52K, Millipore, St. Charles, MO, USA). This assay employs a monoclonal capture antibody (F002; binds all molecular forms of proIAPP) and a monoclonal detection antibody (F025; binds amidated IAPP at its C terminus)^77^ and is 100% cross-reactive to rodent IAPP. To determine active GLP-1, mice were injected with 25 mg kg^−1^ sitagliptin (sc-364620, Santa Cruz Biotechnology, Dallas, USA) 30 min prior to oral glucose loading (2 g kg^−1^ glucose) and blood was collected in tubes containing Diprotin A (H-38250050, Bachem, Bubendorf, Switzerland). Active GLP-1 was assessed by the V-PLEX assay (K1503OD-1, Meso Scale Diagnostics) and serum levels of ghrelin (active), leptin, and PYY (total) were analyzed using the U-PLEX metabolic kit (K152ACL-1, Meso Scale Diagnostics). To determine the ileal content of active GLP-1, 3 cm of the terminal ileum was excised, rinsed with PBS, extracted in 0.18 N HCl in 70% EtOH, and assessed with the kit described above.

### Mass spectrometry to assess steroid hormones

At 11:00, fed mice were anesthetized using isoflurane, blood was collected by cardiac puncture through the skin and the mouse was euthanized by neck dislocation. Blood was incubated at room temperature for 30 min and then centrifuged at 10,000 × g for 20 min at 4 °C. Sera were stored at −80 °C until processing. To precipitate proteins, 100 μl serum was mixed with 100 µl 0.8 M zinc sulfate in water/methanol containing 33 nM internal standards (deuterium-labeled aldosterone (D7), corticosterone (D8), androstenedione (D7) and testosterone (D2). Samples were diluted with 800 μl water followed by incubation in a thermoshaker (10 min, at 4 °C, 1300 rpm) and centrifuged (10 min, 4 °C, 16,000 × g). Then, 950 µl supernatant was transferred to preconditioned Oasis HBL SPE cartridges (1cc, Waters, Milford, MA, USA). Samples were washed with water and methanol/water (10% v/v). Steroids were eluted by adding 750 μl methanol twice, followed by drying for 3 h at 35 °C and reconstitution in 25 μl methanol. Corticosterone, 11-dehydrocorticosterone, 11-deoxycorticosterone, aldosterone, androstenedione, testosterone, and progesterone were analyzed by ultra-high performance liquid chromatography-tandem mass spectrometry using a 1290 Infinity II UPLC coupled to a 6495 triple quadrupole mass spectrometer equipped with a jet-stream electrospray ionization interface (Agilent Technologies, Santa Clara, CA, USA). Analytes were separated using a reverse-phase sub-two-micron column (Acquity UPLC BEH C18, Waters). MassHunter (ver B.10.01, build 10.1.733.0 Agilent Technologies) was used for data acquisition and quantification.

### Glucosuria

To assess glucose concentration in the urine, mice were restrained and urine was collected with a pipette and frozen at −20 °C. Samples were treated with glucose oxidase (G7141, Sigma-Aldrich) and peroxidase (P8250, Sigma-Aldrich), and the resulting H_2_O_2_ concentration was quantified by a colorimetric assay using glucose as standard and employing 4-aminoantipyrine (A4382, Sigma-Aldrich) and sodium 3,5-dichloro-2-hydroxybenzenesulfonate (D4645, Sigma-Aldrich) as coloring reagents. The plate was read at 520 nm on a Synergy H1 Hybrid Multi-Mode Reader (BioTek, Winooski, VT, USA).

### Insulin therapy

One week after the first tamoxifen administration, mice were briefly anesthetized with isoflurane, shaved, and wiped with betadine (Mundipharma, Basel, Switzerland). An 18G needle was used to pinch the skin and a trocar was inserted subcutaneously. Afterward, three insulin pellets (or blanks for controls) were briefly immersed into diluted betadine and transplanted into the flank of the mouse via the previously inserted trocar (LinShin, Toronto, Canada). Pilot studies in streptozotocin-induced C57BL/6N hyperglycemic mice showed that three insulin pellets are needed to normalize blood glucose and that the achieved normoglycemia persisted for about 21 days.

### IAPP sensitivity testing

Mice were single-caged at 5–6 weeks of age and acclimatized to eating from removable custom-made food hoppers. At 10 weeks of age, mice were fasted overnight and intra-peritoneally injected with 20 μg kg^−1^ recombinant rodent IAPP (a gift from Amylin Pharmaceuticals, San Diego, CA, USA), or saline at 9:00. Food hoppers were weighed and returned to the cage 15 min after injection and weighed again 1 and 2 h later. The studies were done in a cross-over design with 1 week wash-out period between tests.

### Implantation of telemetric sensors and indirect calorimetry

Mice were induced at 8 weeks of age and TA-F10 sensors for temperature and activity measurements (Data Sciences International, St. Paul, MN, USA) were implanted intra-peritoneally under isoflurane anesthesia at 10 weeks of age. Thereafter, mice were single-housed. At 11 weeks of age, mice were transported from the University of Basel to the University of Zürich (Tierspital, laboratory of Thomas A. Lutz) and placed in a PhenoMaster open circuit indirect calorimetry system (TSE Systems, Bad Homburg, Germany). The system was calibrated using precision calibration gases prior to use. Air flow rate was 0.41 l min^−1^. Mice were acclimatized to the new environment for 7 days and then oxygen consumption and carbon dioxide production rates were sampled at 20-min intervals for 3 days. From these data, energy expenditure and respiratory exchange ratio were calculated. Physical activity and body temperature were measured every 5 min and analyzed using Dataquest A.R.T. 3.1 software (Data Sciences International).

### Cannula surgeries, intracerebroventricular injections, and refeeding experiments

12–14-week-old female wild-type C57Bl/6N mice were anesthetized by injection of 65 mg kg^−1^ body weight ketamine (Labatec Pharma SA) and 12 mg kg^−1^ body weight xylazine (Streuli Pharma AG) in saline. Viscotears (Lacrinorm, Bausch & Lomb Swiss AG) were applied to the eyes, and the hairs on the top of the skull were shaved. Animals were then affixed to a stereotaxic frame (Kopf). The skin was incised to expose the skull and the periostium was removed by application of acetone. A burr hole was placed at coordinates (*x*: −0.7 mm; *y*: −1.3 mm) from bregma. The guide cannula (C315GS-5/SP, Bilaney consultants) was implanted (*x*: −0.7 mm; *y*: −1.3 mm; *z*: −1.85 mm^78^) to reach the lateral ventricle. To fix the guide cannula in place, cyanoacrylate glue was used and once cured cannula was further fixed using dental acrylic cement (Paladur, Kulzer). Immediately after surgery, postoperative analgesia was performed by i.p. injection of 0.1 mg kg^−1^ body weight Buprenorphine (Bupaq, Streuli Pharma AG). Afterward, 0.3 mg ml^−1^ Buprenorphin was provided via the drinking water for 3 days. Mice remained single-caged and were allowed to recover for at least 2 weeks after surgery. For refeeding studies, mice received 1 µl intracerebroventricular infusions of either saline, 0.4 µU human insulin (Actrapid, Novo Nordisk), or an equimolar amount of human proinsulin (R&D Systems) at 14:00. The mice remained fasted thereafter until food (pre-weighed on a precision balance (K2–20, G&G, Kaarst, Germany)) was once again presented at the onset of the dark cycle (18:00). Any remaining food was weighed 1, 4, 12, 24, and 48 h after refeeding. This was done in a cross-over fashion, although not all mice underwent all treatments as in some occasions guide cannulas were blocked and mice could not be injected.

### Further software

The manuscript was prepared using Microsoft Office version 16.

### Statistical analyses

Data are presented as mean ± SEM. Two groups were compared using the Mann–Whitney *U* test, three or more groups by one-way ANOVA with a Holm–Sidak post hoc test, and data comparing several timepoints by two-way ANOVA with a Holm–Sidak post hoc test. Statistical analyses were conducted with Prism v8.4 (GraphPad Software, San Diego, CA, USA).

### Reproducibility

Bar graphs show an average of all experiments. Representative western blots and FACS blots are shown. Number of independent experiments or in vivo cohorts (defined as pups from different breeding cages and born on different dates) were: Figs. 1A (2), 1B (2), 1C (7), 1D (male 2, female 3), 1E (male 3, female 2), 1F (2), 2A (male 6, female 6), 2B (male 5, female 3), 2C (male 7, female 6), 2D (2), 2E (1), 2F (male 5, female 5), 2G (male 4, female 4), 3A (2), 3B (3), 3C (1), 3D (6), 3E (3), 3F (2), 3G, H (2), 3I–K (2), 3L (male 4, female 4), 4A (male 4, female 5), 4B (4), 4C (6), 4D (2), 4E (1), 4F (2), 4G–J (1), 4K (2), 4L (2), 4M, N (3), 4O (3), 4P (3), 5A, B (3), 5C (samples pooled from various cohorts), 5D (8, some cross-over), and 6 (1); and Supplementary Figs. 1A, B (1), 1C (3), 1D, E (1), 1F (male 4, female 4), 1G (male 3, female 3), 1H (male 4, female 5), 1I (male 5, female 5), 1J (male 5, female 4), 2A (2), 2B (2), 2C (1), 2D (2), 2E (4), 2F (4), 2G–I (2), 2J (1), 2K (2), 2L (2), 2M (1), 3A (3), 3B (4), 3C (4), 3D (2), 3E–H (1), 3I (5), 3J (2), 3K (4), and 3L (2).

### Reporting summary

Further information on research design is available in the Nature Research Reporting Summary linked to this article.

## Supplementary information

Supplementary Information

Peer Review File

Reporting Summary

## Data Availability

The datasets generated and/or analyzed during the current study are available from the corresponding author on reasonable request. Human islet data were extracted from GSE50244. Source Data are provided with this paper.

## Source data

Source Data
