# Supplementary material for: Prohormone convertase 1/3 deficiency causes obesity due to impaired proinsulin processing
Source: Nat Commun. 2022 Aug 13;13:4761. doi: 10.1038/s41467-022-32509-4 (PMC9376086; doi:10.1038/s41467-022-32509-4)
Supplement: Supplementary file 1 — Supplementary Information [file 41467_2022_32509_MOESM1_ESM.pdf]

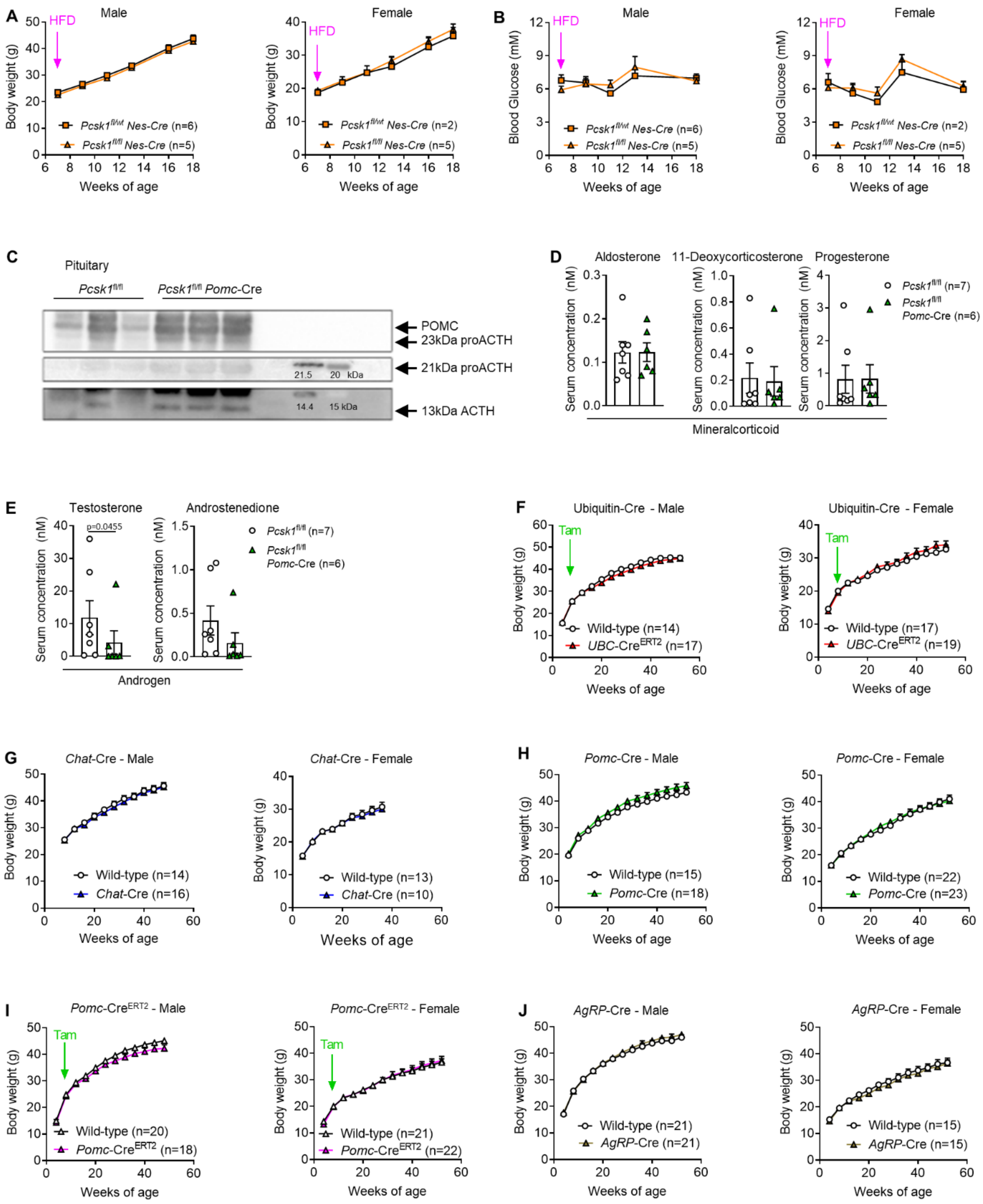

Figure S1. High-fat diet study and body weight development in Cre-only control mice. A-B, Body weight development (**A**) and fed glycemia (8h after lights on) (**B**) in male and female *Pcsk1*$^{fl/wt}$ *Nes*-Cre and *Pcsk1*$^{fl/fl}$ *Nes*-Cre littermate control mice fed a high-fat diet starting at 7 weeks of age. Orange

squares: *Pcsk1*$^{fl/wt}$ *Nes*-Cre, orange triangles: *Pcsk1*$^{fl/fl}$ *Nes*-Cre. **C**, Pituitary protein analysis by western blotting in 16-week-old male *Pcsk1*$^{fl/fl}$ *Pomc*-Cre mice. **D**, Circulating levels of mineralocorticoids in 12-week-old male *Pcsk1*$^{fl/fl}$ *Pomc*-Cre and littermate control mice. **E**, Circulating levels of androgens in 12-week-old male *Pcsk1*$^{fl/fl}$ *Pomc*-Cre and littermate control mice. **F**, Body weight development in *UBC*-Cre^{ERT2} and littermate control mice induced at 8 weeks of age. **G**, Body weight development in *Chat*-Cre and littermate control mice. **H**, Body weight development in *Pomc*-Cre and littermate control mice. **I**, Body weight development in *Pomc*-Cre^{ERT2} and littermate control mice. **J**, Body weight development in *AgRP*-Cre and littermate control mice. Open circles: *Pcsk1*$^{fl/fl}$ (D+E) or wild-type (F-J), colored triangles: Cre (blue: *Chat*-Cre, green: *Pomc*-Cre, purple: *Pomc*-Cre^{ERT2}, golden: *AgRP*-Cre). Data are presented as mean values ± SEM. E was analyzed by a two-sided Mann-Whitney U test. Source data are provided as a Source Data file.

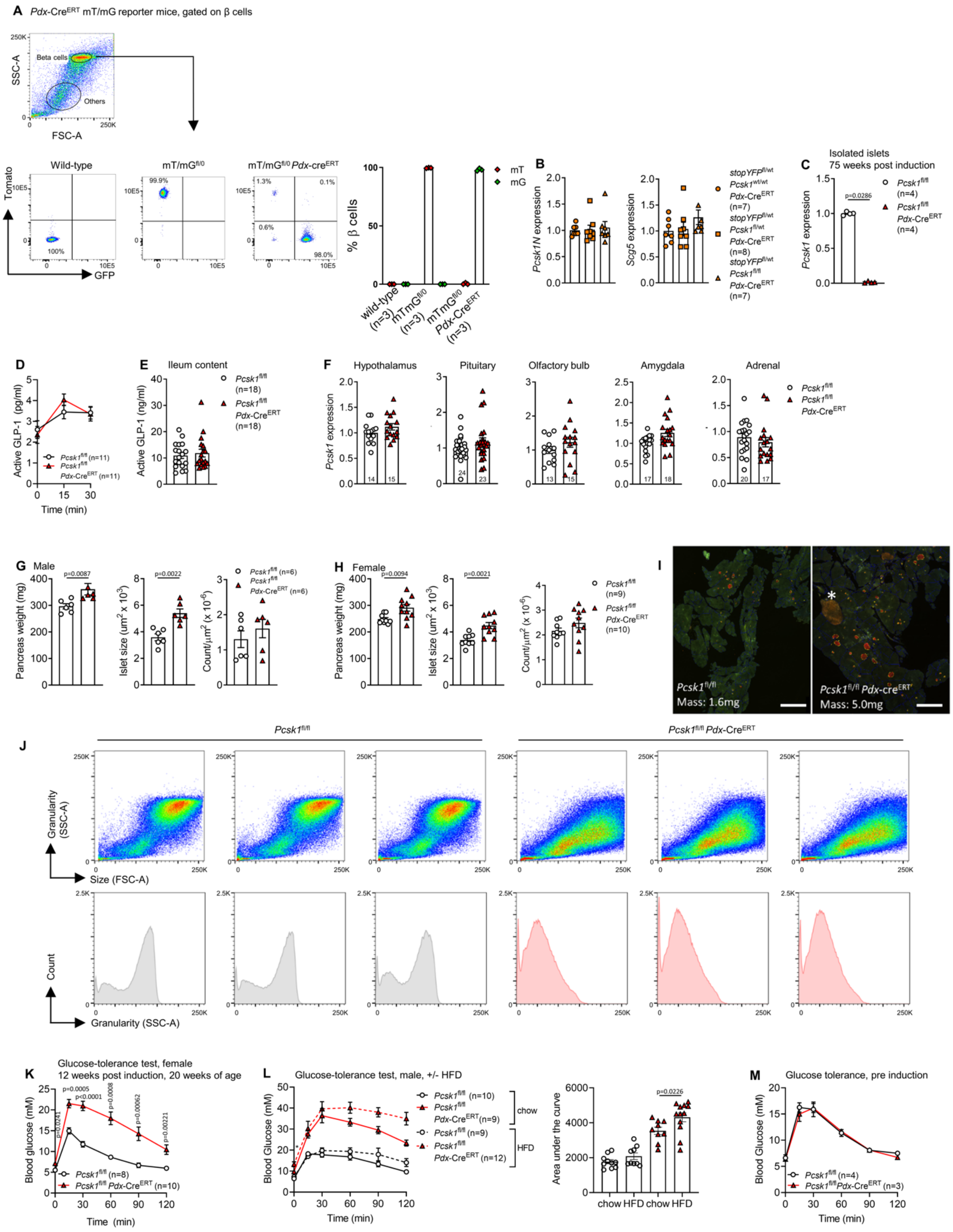

Figure S2. Characterization of *Pcsk1*$^{fl/fl}$ *Pdx*-Cre^{ERT} mice. A, Flow-cytometry analysis of dispersed single cells from islets isolated from 12-week-old mice 4 weeks after induction. Reporter mice were

littermates but the wild-type control mice were age-matched non-littermates. Red diamonds: % beta cells expressing tdTomato, green diamonds: % beta cells expressing GFP. **B**, RNA analysis of FACS-sorted beta cells (YFP+) dispersed from islet isolated from 12-week-old mice 4 weeks after induction. Orange circles: stop*YFP*$^{fl/wt}$ *Pcsk1*$^{wt/wt}$ *Pdx*-Cre^{ERT}, orange squares: stop*YFP*$^{fl/wt}$ *Pcsk1*$^{fl/wt}$ *Pdx*-Cre^{ERT}, orange triangles: stop*YFP*$^{fl/wt}$ *Pcsk1*$^{fl/fl}$ *Pdx*-Cre^{ERT}. **C**, RNA analysis of islets isolated from 83-week-old mice induced at 8 weeks of age. **D**, Circulating levels of active GLP-1 in mice given an oral load of 2 g kg^{-1} glucose with an injection of 25 mg kg^{-1} sitagliptin 30 min prior to glucose loading. **E**, Ileum content of active GLP-1 in mice 5-6 weeks post induction. **F**, RNA analysis of PC1/3-expressing tissues isolated from *Pcsk1*$^{fl/fl}$ *Pdx*-Cre^{ERT} mice 5 weeks post induction. **G**-**H**, Morphometric analysis of pancreata isolated from male and female 20-week-old mice 12 weeks after induction. **I**, Representative pancreas histology (red: insulin, yellow: computer-generated islet outline, green: exocrine pancreas, blue: computer-generated outline of pancreas segments, orange: CD45-positive lymph node (asterisk). Scale bar, 1000 μm. **J**, Flow cytometry analysis of dispersed islet cells from 14-week-old male mice 6 weeks post induction. **K**-**M**, Glucose excursion following an injection of 2 g kg^{-1} glucose after a 6 hour fast in 20-week-old female mice 12 weeks post induction (**K**), in 22-week-old male mice 14 weeks post induction with or without high-fat diet feeding starting at 10 weeks of age (**L**) and in 8-week-old mice without tamoxifen administration (**M**). Open circles: *Pcsk1*$^{fl/fl}$, red triangles: *Pcsk1*$^{fl/fl}$ *Pdx*-Cre^{ERT}. Data are presented as mean values ± SEM. C and E-H were analyzed by a two-sided Mann-Whitney *U* test. D and K-M were analyzed by a mixed-effects analysis with Holm-Sidak's multiple comparisons post hoc test. B+L (area under the curve) were analyzed by a one-way ANOVA with a Holm-Sidak's multiple comparisons post-hoc test. Source data are provided as a Source Data file.

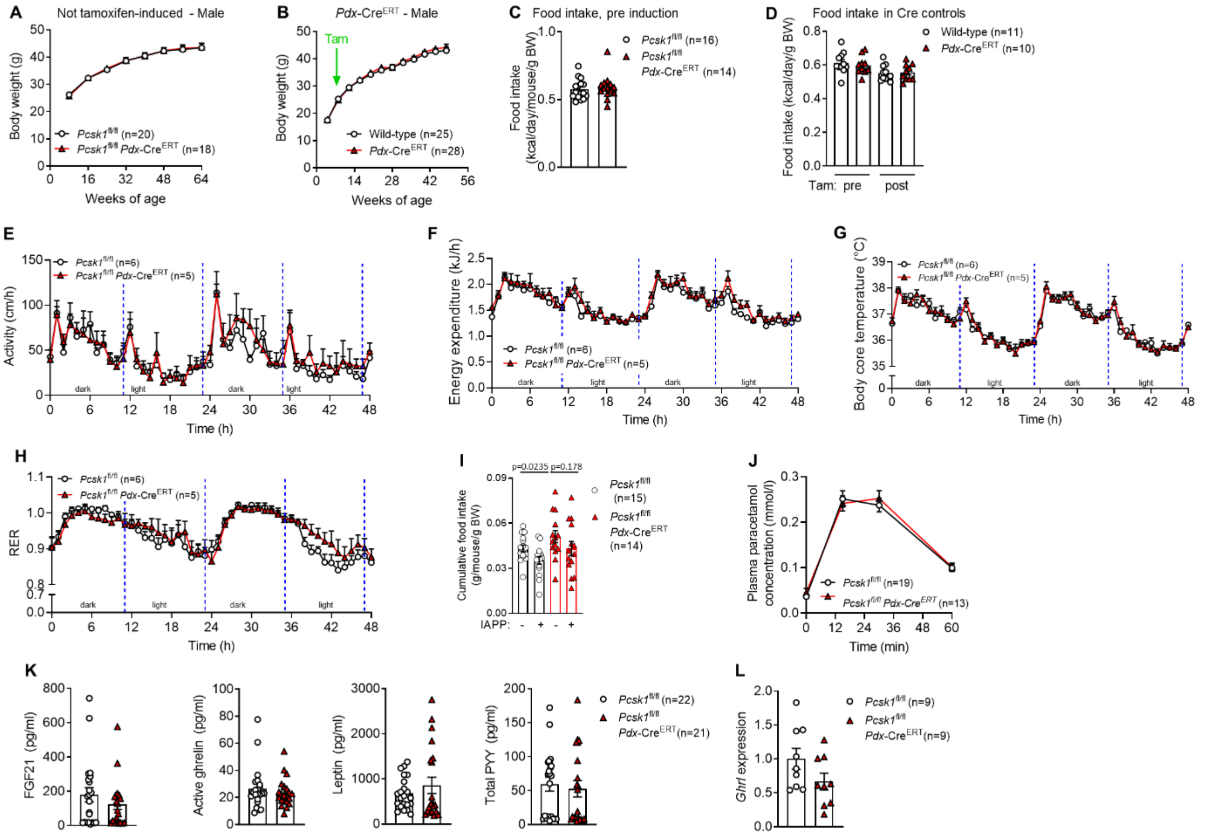

Figure S3 Metabolic assessment of *Pcsk1*$^{fl/fl}$ *Pdx*-Cre^{ERT} mice. A, Body weight development in uninduced male *Pcsk1*$^{fl/fl}$ *Pdx*-Cre^{ERT} and littermate control mice. **B**, Body weight development in male *Pdx*-Cre^{ERT} and littermate control mice induced at 8 weeks of age. Open circles: wild-type, red triangles: *Pdx*-Cre^{ERT}. **C**, Food intake pre induction from 7-8 weeks of age (each dot is the average of a cage with 2-3 mice per cage). **D**, Food intake pre (from 7 to 8 weeks of age) and two weeks post (from 10 to 11 weeks of age) knockout induction in mice expressing *Pdx*-Cre^{ERT} (each dot is the average of a cage with 2-3 mice per cage). Open circles: wild-type, red triangles: *Pdx*-Cre^{ERT}. **E**-**H,** Physical activity (**E**), energy expenditure (**F**), body core temperature (**G**) and respiratory exchange ratio (**H**) at 12 weeks of age 4 weeks post induction. **I**, Cumulative food intake 1 h after refeeding with pre-injection of 20 μg kg^{-1} recombinant IAPP. **J**, Paracetamol appearance in the periphery following oral gavage of 100 mg kg^{-1} paracetamol and 2 g kg^{-1} glucose at 14:00 in 6-hour-fasted mice. **K**, Plasma protein levels in 12-week-old male mice 4 weeks post induction. **L**, Gene expression analysis of stomach samples isolated from 12-week-old male mice 4 weeks post induction. A, C, E-L: Open circles: *Pcsk1*$^{fl/fl}$, red triangles: *Pcsk1*$^{fl/fl}$ *Pdx*-

Cre^{ERT}. Data are presented as mean values ± SEM. I and K+L were analyzed by a two-sided Mann-Whitney *U* test. Source data are provided as a Source Data file.

Gene	Template used	Fw	Rev	Note
Pcsk1	NM_013628.2	TGGATGATGGCTTGGAGTGG	TGCACATCTTGTTCCATGTTTGT	Rev primer spans exon 5-6, designed not to bind recombined floxed Pcsk1 allele
Pcsk2	NM_008792.4	AGAGAGACCCCAGGATAAAGATG	CTTGCCCAGTGTTGAACAGGT	
Cpe	NM_013494.3	CAGCAAGAGGACGGCATCTC	GTCCAACCGCCTCATTACCAT	
Pcsk1N	NM_013892	ACGAGACTCCTGACGTGGA	GCACCTCGGGACCCAAATC	
Scg5	NM_009162.3	CCTTTATGAGAAAATGAAGGG	GGACAGATTTCTTTGCCACA	
Ghrl	NM_021488	TATAAGGAGAAGCCGGTGAGC	TTCTTGGATTCCTTTCTCTGCTGG	
Act	NM_007393.4	GGCTGTATTCCCCTCCATCG	CCAGTTGGTAACAATGCCATGT	
Gapdh	NM_001289726.1	AGGTCGGTGTGAACGGATTTG	TGTAGACCATGTAGTTGAGGTCA	
18S	NR_003278.3	GGGAGCCTGAGAAACGGC	GGGTCGGGAGTGGGTAATTT	

Table S1. Primer sequences used for qPCR

Name	Art Nr	Source	clone	dilution	Identifier	FACS/IHC	Note
anti-CD16/CD32	14-0161	Thermo Fisher Scientific	93	1:100	RRID: AB_467134	FACS	
anti-CD45-APC	17-0451-83	eBioscience	30-F-11	1:100	RRID: AB_469393	FACS	
guinea pig anti-insulin	A0564	Thermo Fisher (Dako)	poly	1:2000	RRID: AB_10013624	IHC/WB	Discontinued
rat anti-CD45	550539	BD Biosciences	30-F-11	1:200	RRID: AB_2174426	IHC	
anti PC1/3	AB10553	Millipore	poly	1:100	RRID: AB_1977441	IHC	Discontinued
Alexa Fluor 647-conjugated goat anti–guinea pig IgG	A21450	Thermo Fisher Scientific		1:1000	RRID: AB_141882	IHC	
Alexa Fluor 555-conjugated goat anti-rat IgG	A21434	Thermo Fisher Scientific		1:1000	RRID: AB_141733	IHC	
anti PC1/3 N-term	1BF	Iris Linderg		1:1000		WB	
anti PC1/3 N-term	1BF	Richard E. Mains		1:1000		WB	affinity-purified batch. Originally provided by Iris Lindberg to Richard E. Mains
anti actin	A1978	Sigma-Aldrich		1:2000	RRID: AB_476692	WB	
anti ACTH	A-1A12	Anne White		1:1000 (stock 1mg/ml)		WB	
rabbit anti mouse Iapp	T-4145	BMA Biomedicals (Peninsula)		1:1000	RRID: AB_518030	WB	Discontinued
anti Iapp mature	F025	Amylin Pharmaceuticals		1:1000		WB	Not commercially available
rabbit anti AKT	4685S	Cell Signaling	11E7	1:2000	RRID: AB_2225340	WB	
rabbit anti pAKT Ser473	9271S	Cell Signaling		1:2000	RRID: AB_329825	WB	
rabbit anti Hsp90	4874S	Cell Signaling		1:1000	RRID: AB_2121214	WB	
goat-anti-mouse HRP	SC-2005	Santa Cruz		1:2000	RRID: AB_631736	WB	Discontinued
goat-anti-mouse HRP	SC-516102	Santa Cruz		1:2000	RRID: AB_2687626	WB	
rabbit-anti-guinea-pig HRP	P0141	Thermo Fisher (Dako)		1:2000		WB	Discontinued
goat-anti-rabbit HRP	A16104	Invitrogen		1:2500	RRID: AB_2534776	WB	

rabbit-anti-UCP1	ab10983	Abcam		1:1000	RRID: AB_2241462	WB	

Table S2. Antibodies used for histology and western blotting
