# Supplementary material for: Prohormone convertase 1/3 deficiency causes obesity due to impaired proinsulin processing
Source: Nat Commun. 2022 Aug 13;13:4761. doi: 10.1038/s41467-022-32509-4 (PMC9376086; doi:10.1038/s41467-022-32509-4)
Supplement: Supplementary file 3 — Reporting Summary [file 41467_2022_32509_MOESM3_ESM.pdf]

nature portfolio

Corresponding author(s): Daniel T. Meier

Last updated by author(s): Jul 19, 2022

Reporting Summary

Nature Portfolio wishes to improve the reproducibility of the work that we publish. This form provides structure for consistency and transparency in reporting. For further information on Nature Portfolio policies, see our Editorial Policies and the Editorial Policy Checklist.

Statistics

For all statistical analyses, confirm that the following items are present in the figure legend, table legend, main text, or Methods section.

n/a	Confirmed	
☐	☒	The exact sample size (*n*) for each experimental group/condition, given as a discrete number and unit of measurement
☐	☒	A statement on whether measurements were taken from distinct samples or whether the same sample was measured repeatedly
☐	☒	The statistical test(s) used AND whether they are one- or two-sided<br>*Only common tests should be described solely by name; describe more complex techniques in the Methods section.*
☒	☐	A description of all covariates tested
☐	☒	A description of any assumptions or corrections, such as tests of normality and adjustment for multiple comparisons
☐	☒	A full description of the statistical parameters including central tendency (e.g. means) or other basic estimates (e.g. regression coefficient) AND variation (e.g. standard deviation) or associated estimates of uncertainty (e.g. confidence intervals)
☐	☒	For null hypothesis testing, the test statistic (e.g. *F*, *t*, *r*) with confidence intervals, effect sizes, degrees of freedom and *P* value noted<br>*Give P values as exact values whenever suitable.*
☒	☐	For Bayesian analysis, information on the choice of priors and Markov chain Monte Carlo settings
☒	☐	For hierarchical and complex designs, identification of the appropriate level for tests and full reporting of outcomes
☒	☐	Estimates of effect sizes (e.g. Cohen's *d*, Pearson's *r*), indicating how they were calculated

Our web collection on statistics for biologists contains articles on many of the points above.

Software and code

Policy information about availability of computer code

Data collection	Dataquest A.R.T. ver3.1 software (Data Sciences International), MassHunter ver B.10.01 build 10.1.733.0 (Agilent Technologies), NIS Elements ver 5.21.03 (Nikon), FlowJo ver10.6.1 (FlowJo LLC),
Data analysis	Prism ver8 (GraphPad), Excel ver16.62 (Microsoft), Fiji/ImageJ ver2 (NIH), ilastik ver 1.3.3post3 (HCI), custom script ImageJ (Unibas Microscopy Core Facility)

For manuscripts utilizing custom algorithms or software that are central to the research but not yet described in published literature, software must be made available to editors and reviewers. We strongly encourage code deposition in a community repository (e.g. GitHub). See the Nature Portfolio guidelines for submitting code & software for further information.

Data

Policy information about availability of data

All manuscripts must include a data availability statement. This statement should provide the following information, where applicable:

- Accession codes, unique identifiers, or web links for publicly available datasets
- A description of any restrictions on data availability
- For clinical datasets or third party data, please ensure that the statement adheres to our policy

Data availability statement. The datasets generated during and/or analysed during the current study are available from the corresponding author on reasonable

request. Human islet data were extracted from GSE50244 [https://www.ncbi.nlm.nih.gov/geo/query/acc.cgi?acc=GSE50244]. Source data are provided with this paper.

Human research participants

Policy information about studies involving human research participants and Sex and Gender in Research.

Reporting on sex and gender	NA
Population characteristics	NA
Recruitment	NA
Ethics oversight	NA

Note that full information on the approval of the study protocol must also be provided in the manuscript.

Field-specific reporting

Please select the one below that is the best fit for your research. If you are not sure, read the appropriate sections before making your selection.

☒ Life sciences ☐ Behavioural & social sciences ☐ Ecological, evolutionary & environmental sciences

For a reference copy of the document with all sections, see nature.com/documents/nr-reporting-summary-flat.pdf

Life sciences study design

All studies must disclose on these points even when the disclosure is negative.

Sample size	Sample size were based on experience with the methods used (PMID: 32080229, 28355570, 28092375). Power calculations were used to guide animal numbers.
Data exclusions	No data was excluded.
Replication	As a general rule, experiments were performed in at least 3 independent cohorts (litters from a different breeding pair, experiment done at a different point in time). All attempts at replication were successful.
Randomization	Cohorts of experimental animals and their littermate controls were allocated to different analysis randomly. Knockout mice and their matching littermate controls sacrificed 4- and 12 weeks post induction shown in Figure 4D were chosen randomly. Data shown in Figures 4O, 5A-B, 5D were generated by random allocation of animals into experimental groups. For all other experiments, experimental allocation was determined by genotype (knockout vs control).
Blinding	Glucose-tolerance tests were done without knowing the genotype of the mice. Studies involving manual scoring/analysis were conducted in a blinded manner. Studies involving injecting substances could not be done in a blided manner.

Reporting for specific materials, systems and methods

We require information from authors about some types of materials, experimental systems and methods used in many studies. Here, indicate whether each material, system or method listed is relevant to your study. If you are not sure if a list item applies to your research, read the appropriate section before selecting a response.

Materials & experimental systems

n/a	Involved in the study	
☐	☒	Antibodies
☒	☐	Eukaryotic cell lines
☒	☐	Palaeontology and archaeology
☐	☒	Animals and other organisms
☒	☐	Clinical data
☒	☐	Dual use research of concern

Methods

n/a	Involved in the study	
☒	☐	ChIP-seq
☐	☒	Flow cytometry
☒	☐	MRI-based neuroimaging

Antibodies

Antibodies used	Company names, catalogue numbers and dilutions can be found in the methods section and Table S1
Validation	- PC1/3 antibody 1BF (Western blotting, mouse tissue) was validated in this manuscript using KO tissue: Fig. 1B, 3B<br>- PC1/3 antibody AB10553 (histology, mouse tissue) was validated in this manuscript using KO tissue: Fig. 3C<br>- Iapp antibody T-4145 and F025 (Western blotting, mouse tissue) was validated in this manuscript using KO tissue: Fig. 4M, 4N<br>- ACTH antibody was characterized and validated (Western blotting, mouse) in Crosby et al (PMID: 284055)<br>- CD16/CD32 antibody 14-0161 (flow, mouse splenocytes): https://www.thermofisher.com/antibody/product/CD16-CD32-Antibody-clone-93-Monoclonal/14-0161-82<br>- CD45 antibody 17-0451-83 (flow, mouse bone marrow cells): https://www.thermofisher.com/antibody/product/CD45-Antibody-clone-30-F11-Monoclonal/17-0451-82<br>- Insulin antibody A0564 (Western blotting, mouse islet lysate) was validated in this manuscript: Fig. 1F, 3D<br>- Insulin antibody A0564 (Histology, mouse pancreas) was validated in this manuscript: Fig. 3C, S2I<br>- CD45 antibody 550539 (histology, mouse spleen): https://www.bdbiosciences.com/en-sg/products/reagents/microscopy-imaging-reagents/immunohistochemistry-reagents/purified-rat-anti-mouse-cd45.550539<br>- Actin antibody A1978 (Western blotting, human lung cell line): https://www.sigmaaldrich.com/CH/de/product/sigma/a1978<br>- AKT antibody 4685S (CHO cells, hamster, siRNA): https://www.cellsignal.com/products/primary-antibodies/akt-antibody/9272<br>- pAKT antibody 9271S (Western blotting, 3T3 cells, mouse): https://www.cellsignal.com/products/primary-antibodies/phospho-akt-ser473-antibody/9271?site-search-type=Products&N=4294956287&Ntt=9271s&fromPage=plp&_requestid=75505<br>- HSP90 antibody 4874S (Western blotting, 3T3 cells, mouse): https://www.cellsignal.com/products/primary-antibodies/hsp90-antibody/4874?site-search-type=Products&N=4294956287&Ntt=4874s&fromPage=plp&_requestid=75829<br>- UCP antibody ab10983 (Histology mouse brown adipose tissue; Western blotting rat brown adipose tissue): https://www.abcam.com/ucp1-antibody-ab10983.html

Animals and other research organisms

Policy information about studies involving animals; ARRIVE guidelines recommended for reporting animal research, and Sex and Gender in Research

Laboratory animals	Wild-type or genetically modified mice (mus musculus) were used. All mice were on a C57BL/6N genetic background (backcrossed to C57BL/6N for > 10 generations). Most experiments were done with male and female mice. All controls were littermates. In inducible models, the control mice also received tamoxifen. Information about sex and animal numbers can be found in the figure, figure legends and methods. Age was 4-75 weeks of age (specific experiment see figures and figure legends). Genetically modified mouse lines used were:<br>B6N-Pcsk1tm1Boe, UBC-Cre B6.Cg-Ndor1Tg(UBC-cre/ERT2)1Ejb, B6.Cg-Tg(Nes-Cre)1Kln, B6.FVB-Tg(Pomc1-Cre)16Lowl, B6.STOCK-Agrptm1(cre)Lowl, B6;129S6-Chattm2(cre)Lowl, B6.129(Cg)-Gt(ROSA)26Sortm4(ACTB-tdTomato,-EGFP)Luo, B6.129X1-Gt(ROSA)26Sortm1(EYFP)Cos, B6N.STOCK-Gt(ROSA)26Sortm1(FLP1)Dym, B6.STOCK Tg(Pdx1-Cre/Esr1*)Dam, B6.129P2-Iapptm1Sgm, B6.Cg-Tg(Pomc-Cre)ERT2Jke
Wild animals	This study does not contain wild animals
Reporting on sex	Most experiments were done in both sexes and reported separately.
Field-collected samples	This study does not contain field-collected samples
Ethics oversight	Ethical overseight and approval by the cantonal veterinary office of Basel city (approval number 2511 and 3045)

Note that full information on the approval of the study protocol must also be provided in the manuscript.

Flow Cytometry

Plots

Confirm that:

☒ The axis labels state the marker and fluorochrome used (e.g. CD4-FITC).

☒ The axis scales are clearly visible. Include numbers along axes only for bottom left plot of group (a 'group' is an analysis of identical markers).

☒ All plots are contour plots with outliers or pseudocolor plots.

☒ A numerical value for number of cells or percentage (with statistics) is provided.

Methodology

Sample preparation	Details can be found in the methods section. Briefly, pancreatic islets were isolated by collagenase digestion. Islets were dispersed using Trypsin/EDTA. Cells were then labeled and separated and/or analysed by flow-cytometry.
Instrument	CytoFLEX flow cytometer, BD Fortessa, BD SORPAria 3, BD FACSAria 3, BD Influx cell sorter

Software	FlowJo
Cell population abundance	Beta cells are the most abundant cell type (approximately 80%) of all islet cells. Cell purity and identity was analysed by qPCR (see results section)
Gating strategy	A detailed illustration of the gating strategy can be found in the supplemental information

☒ Tick this box to confirm that a figure exemplifying the gating strategy is provided in the Supplementary Information.
